# Fused Pyrroles in Cholestane and Norcholestane Side Chains: Acaricidal and Plant Growth-Promoting Effects

**DOI:** 10.3390/molecules27238466

**Published:** 2022-12-02

**Authors:** María G. De los Santos, Marcos Cua-Basulto, Anallely Huepalcalco, Wendy Delit, Jesús Sandoval-Ramírez, Adolfo López-Torres, Esaú Ruiz-Sánchez, María A. Fernández-Herrera

**Affiliations:** 1 Departamento de Física Aplicada, Centro de Investigación y de Estudios Avanzados, Unidad Mérida, Km 6 Antigua Carretera a Progreso, Apdo, Postal 73, Cordemex, Mérida 97310, Yucatán, Mexico; 2 División de Estudios de Posgrado e Investigación, Tecnológico Nacional de México Campus Conkal, Conkal 97345, Yucatán, Mexico; 3 Facultad de Ciencias Químicas, Benemérita Universidad Autónoma de Puebla, Ciudad Universitaria, Puebla 72570, Puebla, Mexico; 4 Centro de Investigaciones Científicas, Instituto de Química Aplicada, Universidad del Papaloapan, Circuito Central Num. 200, Col. Parque Industrial, Tuxtepec 68301, Oaxaca, Mexico

**Keywords:** fused pyrroles, microwave synthesis, norcholestanes, acaricide activity, growth-promoting activity

## Abstract

Herein, we describe the synthesis and characterization of fused pyrroles in cholestane and norcholestane side chains derived from kryptogenin and diosgenin, respectively. Both conventional and microwave heating techniques were used to synthesize the steroidal pyrroles from primary amines, with the microwave method producing the highest yields. In particular, the norcholestane pyrroles were tested as acaricides against the two-spotted spider mite (*Tetranychus urticae* Koch) under laboratory conditions and as plant growth promoters on habanero pepper (*Capsicum chinense* Jacq) under greenhouse conditions.

## 1. Introduction

Pyrroles are among the most outstanding heterocycles and are constituents in numerous natural products, synthetic pharmaceuticals, and high-value materials [1,2,3,4]. This has encouraged the development of new pathways for synthesizing pyrrole rings and the appearance of new pyrrole-based supramolecular complexes. Some of the most well-known naturally occurring pyrrole derivatives include porphyrins [5], chlorophylls [6], and bile pigments [7].

The Paal–Knorr reaction [8,9,10], in which 1,4-dicarbonyl systems are converted into pyrroles by an acid-mediated dehydration cyclization in the presence of a primary amine, is a frequently used method for making pyrroles. The 1,4-dicarbonyl framework supplies the pyrrole’s four carbons, and the amine provides the nitrogen. The main limitations of this reaction are typically the harsh reaction conditions required for cyclization; as a result, pyrrole synthesis is still challenging.

**Figure 1:**
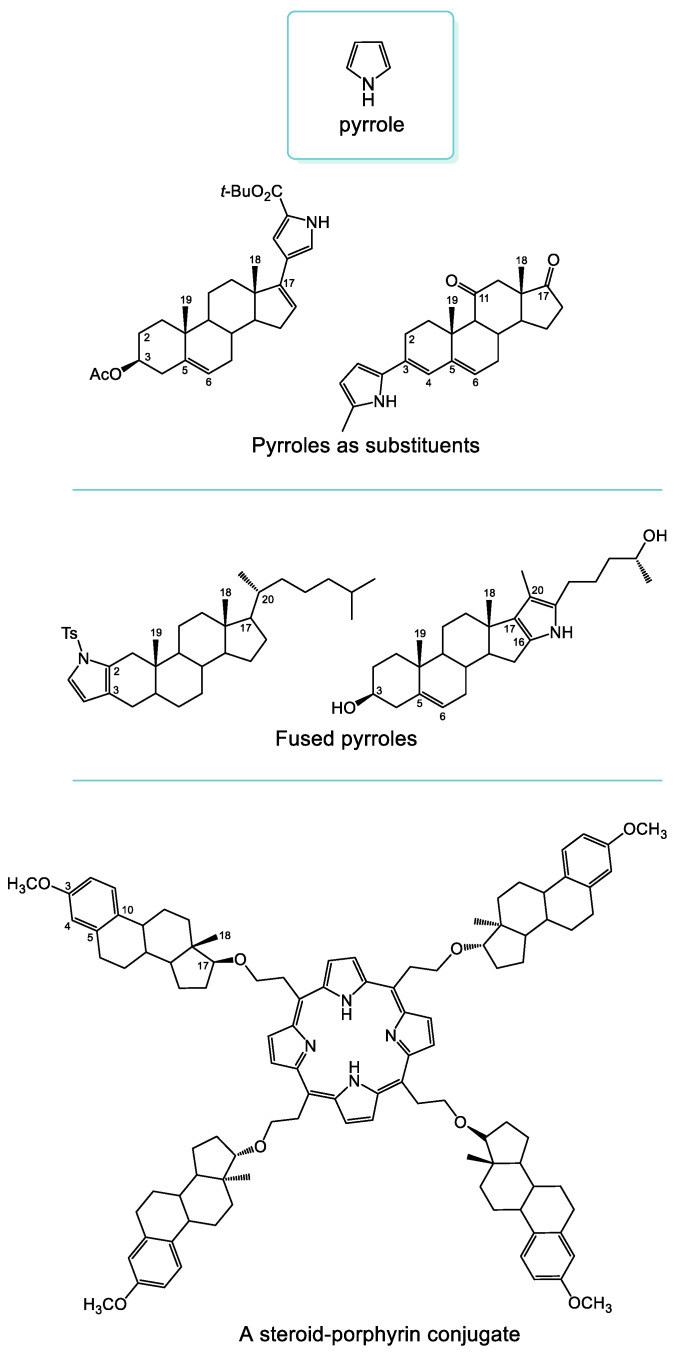
Pyrrole rings attached to steroidal frameworks at different positions.

Pyrroles can be incorporated into steroidal structures as substituents in either the side chain or the core (Figure 1). In order to prepare vinylic pyrroles from nitrosteroids, Barton et al. [11] established a practical method for synthesizing pyrroles from nitroolefins. In one step, Zaitsev et al. [12] used superbasic catalytic systems and the Trofimov reaction to bind a steroid fragment to a pyrrole ring from steroid ketone oximes and acetylene. Koivukorpi and co-workers [13] described the synthesis and characterization of 5β-cholane skeletons supporting pyrrole groups at C-3 and C-24. Recent investigations on steroidal oxadiazole, pyrrole, and pyrazole derivatives of cholesterol were reported by Shamsuzzaman et al. [14], including their synthesis, characterization, and anticancer effects.

Metz [15] reported the synthesis of pyrrolosteroidal dienes by adding 2-methylpyrrole to adrenosterone, progesterone, and 17α-acetoxyprogesterone at the C-3 position. The synthesis of spiroannulated oligopyrrole macrocycles, calixpyrroles, and steroid–porphyrin conjugates also represent a study field, as the physicochemical and aggregation properties of these systems are crucial in supramolecular chemistry [16,17,18,19,20].

However, pyrroles fused to the steroidal core are less frequent. One of the early contributions to the Paal–Knorr reaction of kryptogenin (**1**) was made by Mueller and Jiu [21], under harsh conditions. Recently, Davies and co-workers [22] converted cyclic ketones to 2,3-fused pyrroles using a series of substrates and applied the methodology to complex frameworks such as a cholestene vinyl triflate to fuse the pyrrole heterocycle to ring A at positions 2 and 3 (see fused pyrroles in Figure 1).

Our research group is currently interested in synthesizing new steroidal cores via the side-chain transformation of steroidal sapogenins. In steroidal systems, the Paal–Knorr reaction has not been studied since the 1960s study of Mueller and Jiu. Herein, we revisited the Paal–Knorr reaction in steroids and describe the synthesis and characterization of fused pyrroles in cholestane and norcholestane side chains derived from kryptogenin and diosgenin, respectively, under conventional and microwave-assisted heating.

We decided to investigate the usefulness of norcholestane pyrroles as acaricides and their influence as growth regulators because we hypothesized their potential for future use in agrochemical applications. Regarding acaricidal activity, we considered that the uncontrolled use of non-selective pesticides and the high reproductive capacity of *Tetranychus urticae* Koch had significantly accelerated the emergence of pest resistance. In order to ensure efficient mite control, those facts resulted in an increase in the rate and amount of pesticide applications [23]. As a result, agriculture-derived products frequently contain high concentrations of pesticide residues that are harmful to human health. Due to this, it has been reported in multiple studies that focus on developing better alternatives, such as acaricides, for managing phytophagous mites [24,25,26,27]. Compounds containing pyrrole rings in their structure can potentially be alternatives for controlling phytophagous mites and insects, e.g., chlorfenapyr [26,28], the first compound of this class to be registered for mite control, derived from the naturally occurring dioxapyrrolomycin [28,29,30]. Because the two-spotted spider mite, *T. urticae* [31], is an extremely polyphagous mite and one of the most important crop pests worldwide [32], we evaluated the norcholestane pyrroles on their acaricidal activity under laboratory conditions. Additionally, we investigated the impact of norcholestane pyrroles as plant growth regulators on the vegetative development of the habanero pepper (*Capsicum chinense* Jacq) under greenhouse conditions, taking into account the history of the use of various steroidal compounds [23,24], such as brassinosteroids [25], to promote plant growth, root development, and the production of flowers and fruits. In particular, there are not many reports of pyrrole-containing compounds or pyrrole precursors being investigated for promoting plant development [33,34].

Therefore, in this manuscript, we investigate the synthesis of fused pyrroles in cholestane and norcholestane side chains and evaluate their acaricidal and growth-promoting effects on plants.

## 2. Results and Discussion

### 2.1. Chemical Synthesis

**Scheme 1:**
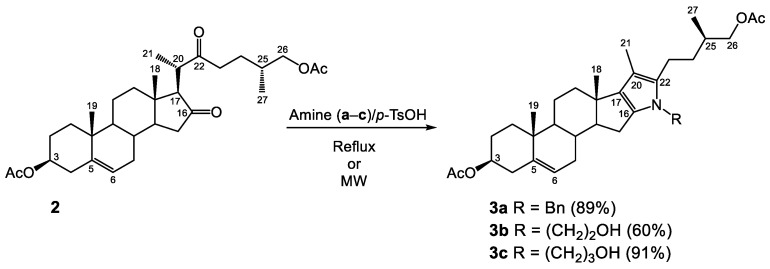
Pentasubstituted pyrroles fused in a cholestane side chain.

Earlier experiments of ring closure to produce a pyrrolidino-steroid were launched from the 1,4-diketone system of 16,22-dioxo-5*β*-cholestan-26-oic acid and through the hydrogenation of the dioxime derivative. Uhle et al. [35,36] improved the formation of a pyrrolidino-steroid from the reduction of the 2,4-dinitrophenylhydrazone attached at C-16 of **1**. Subsequently, Mueller and Jiu [21] reported on the synthesis of tetrasubstituted pyrroles from **1** by direct condensation under harsh conditions using an ethanol/ammonia solution at 135 °C and 480 psi for 4 h in an autoclave. Pentasubstituted pyrroles were produced using an acetic acid/aqueous methylamine solution, heated at 130 °C for 90 min and left overnight. Herein, we first revisit the Paal–Knorr reaction on **1** to further apply this approach to norcholestane derivatives obtained from diosgenin. Two different heating techniques were explored for synthesizing the steroidal pyrroles through the Paal–Knorr reaction: conventional heating at reflux and microwave (MW) heating. Three primary amines, benzylamine (**a**), ethanolamine (**b**), and propanolamine (**c**), were chosen as sources of the nitrogen atom. Kryptogenin (**1**) was diacetylated, under standard conditions (Ac_2_O/Py) [37], given that the Paal–Knorr reaction does not proceed if the hydroxyl at C-26 is unprotected and an intramolecular cyclization of the hydroxyl at C-26 and the carbonyl at C-22 occurs in the side chain above room temperature. The 3,26-diacetate of kryptogenin (**2**) and benzylamine (**a**) were selected to determine the best reaction conditions under a series of solvents at reflux and *p*-TsOH as catalyst (see Scheme 1 and Table 1). The experiments listed in Table 1 show the best yield obtained at reflux with each solvent. All the reactions were carried out for several hours and monitored by thin-layer chromatography (TLC) every 30 min. The times indicated are those with the highest yields. 

Regarding DMF, it was observed that the substrate was decomposed at reflux, and the temperature was decreased until an optimum value was obtained (150 °C) in which no decomposition products were observed. The best performance was achieved using toluene. Therefore, under the reaction conditions of entry 3, the three primary amines (**a**–**c**) successfully yielded pyrroles **3a**–**c** at reflux.

The next step consisted of improving the reaction conditions by employing microwave-assisted synthesis. The use of MW in organic synthesis has been widely documented, with benefits such as sample reduction, reduced reagent consumption, process automation, and improved yields [38,39,40]. On this basis, we explored the Paal–Knorr reaction of kryptogenin 3,26-diacetate and the amines **a**–**c** under MW heating. Our initial objective was to replace toluene with a green solvent. Of course, toluene is not even close to being the first choice for MW, but none of the solvents tested (acetonitrile, ethanol, ethyl acetate, xylene, DMF) provided better yields in the MW reactor. A misconception about microwave heating is that microwaves can only benefit experiments involving polar solvents. The benefits of microwave heating can be harnessed despite the dielectric characteristics of a given solvent. Most reactions involve polar or ionic species with which microwave energy can interact directly, even if the solvent does not absorb efficiently. As toluene presents a dielectric constant of 2.38, it is not an efficient solvent to absorb microwaves and undergo heating, so SiC vials were used to conduct these reactions. The reaction time on MW dropped from 5 h to 80 min, and all yields were improved.

After optimizing the reaction conditions for the synthesis of **3a**–**c**, a 1,4-dicarbonyl system was constructed from diosgenin (**4**) through a series of reactions at the side chain. The cleavage of rings E and F of the spiroketal was conducted through a Lewis acid-catalyzed acetolysis to get the cholestane skeleton bearing a hydroxyl group at C-26 (compound **5**). Oxidation of alcohol **5** with freshly prepared pyridinium chlorochromate (PCC) in dichloromethane (DCM) [41] yielded aldehyde **6**. The spectral data of **5** and **6** matched earlier reports [42,43]. Having the 1,5-dicarbonyl substrate **6** in hand, we performed a C-C bond cleavage between C-25 and C-26 to construct the 1,4-dicarbonyl system using the methodology reported by Tiwari et al. [44]. This procedure involves the addition of an electron-rich amine, the 3,4,5-trimethoxyaniline, to form a Schiff base leading to the subsequent formation of an enamine and finally to the cleavage of the C-C bond (carbon–aldehyde carbonyl bond C-C=O). Hamid et al. reported a variant of the methodology of Tiwari et al. to cleave an aldehyde C-C bond in a furostane structure’s side chain [45], but using ethanol instead of toluene. Indeed, by using compound **6** as starting material, the reaction with toluene did not provide good yields, and the reaction with ethanol led to a similar yield to the one reported for furostanes by Hamid et al. This reaction step can also be accomplished with methanol, isopropanol, and acetonitrile as solvents, albeit in lower yields. Therefore, this procedure provided the norcholestane compound **7** (the 1,4-dicarbonyl system) in good yield.

**Scheme 2:**
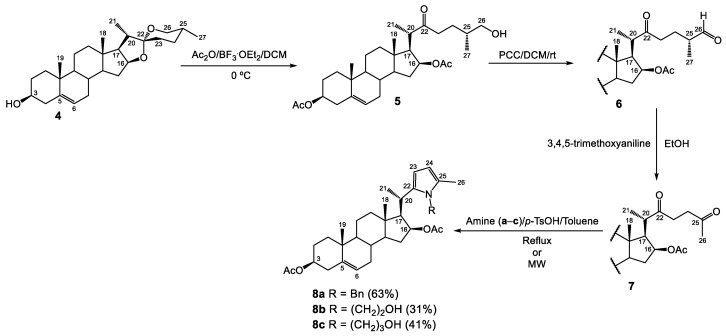
Trisubstituted pyrroles fused in a norcholestane side chain obtained from diosgenin.

Following the established reaction conditions for kryptogenin derivatives, the Paal–Knorr reaction was performed using the 1,4-dicarbonyl system **7** and the primary amines **a**–**c** chosen for kryptogenin assays. The experiments yielded the fused norcholestane pyrroles **8a**–**c** (Scheme 2) under reflux and MW heating.

### 2.2. NMR Characterization

Table 2 shows selected ^1^H NMR chemical shifts for **3a**–**c** and **8a**–**c**. For **3a**–**c**: **3a** exhibits an effect of the presence of benzyl by slightly altering signals such as H-26 and the CH_3_-27 towards lower frequencies (0.1–0.2 ppm) when compared to **3b** and **3c**. CH_3_-18 and CH_3_-21 are observed towards higher frequencies (0.02–0.03 ppm). The signals of H-3, H-6, and CH_3_-19 remain significantly unchanged. For **8a**–**c**: **8a** also shows an effect of the presence of benzyl where H-20, CH_3_-21, and CH_3_-26 are observed toward lower frequencies, approximately 0.4 ppm for 21, 0.1 ppm for 26, and 0.6 ppm for 20, relative to **8b** and **8c**. The signals of H-3, H-6, H-16, CH_3_-18, and CH_3_-19 remain practically unchanged. The signals for H-23 and H-24 of pyrroles **8a**–**c** corroborate the formation of the heterocycle; these appear at 5.78, 5.75, and 5.85 ppm, respectively.

Regarding ^13^C NMR, Table 3 shows selected chemical shifts for **3a**–**c** and **8a**–**c**. For compounds **3a**–**c**, the pyrrole ring takes up positions 16, 17, 20, and 22, and, as expected, its chemical shifts are found in the range of 108 to 135 ppm, which corroborates the embedded ring in the cholestane structure. For the embedded pyrrole rings in the norcholestane structure of **8a**–**c**, the ring takes up positions 22, 23, 24, and 25, and its chemical shifts are observed in the 102 to 138 ppm region. Those chemical shifts also support the formation of norcholestane pyrroles.

**Figure 2:**
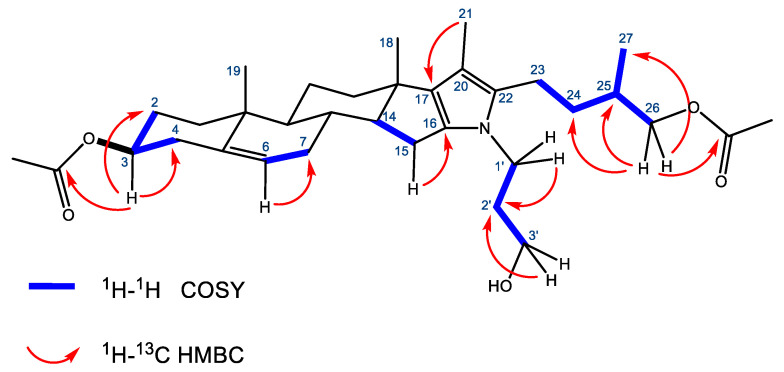
Key ^1^H-^1^H COSY and ^1^H-^13^C HMBC correlations using **3c** as a model.

A combination of COSY, HSQC, and HMBC experiments (see supplementary materials) helped to complete the ^1^H and ^13^C NMR assignments of the cholestane and norcholestane pyrroles **3a**–**c** and **8a**–**c**. Figure 2 shows a representative scheme of key COSY and HMBC correlations using **3c** as a model.

### 2.3. Biological Evaluation

#### 2.3.1. Acaricidal Activity against the Two-Spotted Spider Mite (*Tetranychus urticae* Koch)

The acaricidal activity of **8a**–**c** was evaluated on adults and eggs. For adults, **8a** and **8b** induced significant mortality at 24 h after spraying. At 48 and 72 h, all compounds resulted in significant mortality. Notably, at 72 h, **8c** produced the highest effect (Table 4). For eggs, **8b** and **8c** caused significant mortality. All compounds had a modest effect on adults. However, **8c** showed a highly lethal effect on eggs.

The residuality of a new pesticide for plant protection is critical to consider when assessing its activity. In this sense, we can assume that pyrrole compounds have a fear residual time based on other studies showing that pyrroles, such as chlorfenapyr, are active one to three weeks after application [46,47,48]. However, it is worth noting that plant species, age, and environmental conditions can influence such time. 

#### 2.3.2. Plant Growth Evaluation on Habanero Pepper (*Capsicum chinense* Jacq)

The activity of the norcholestane pyrroles **8a**–**c** on vegetative growth in habanero peppers (*C. chinense*) was evaluated under greenhouse conditions. In these experiments, the variables were collected 15 days after compounds **8a**–**c** were directly sprayed on plants. To determine the sampling dates, the response time of norcholestane pyrroles was considered. Treatments had no meaningful effect on plant growth (plant height and number of leaves per plant). However, regarding biomass accumulation (Table 5), root dry biomass was significantly higher in plants treated with compound **8c** (0.15 g/plant) than for control plants (0.10 g/plant). Plant root system growth and development require coordinated endogenous and environmental signal regulation. Previous studies have shown that plant root growth and development are intrinsically linked to phytohormones [49]. This result can be further explored to determine the optimal concentration of **8c** for root elongation in habanero pepper plants, as it would lead to the development of stronger and more vigorous plants and a better understanding of the role of **8c** as phytohormone.

## 3. Materials and Methods

### 3.1. General Remarks

Commercially available materials purchased from Merck were used as received. Diosgenin and kryptogenin were purified via column chromatography and solvents via distillation before use. The ^1^H and ^13^C NMR spectra were recorded in CDCl_3_ on an Agilent DD2 600 spectrometer (^1^H NMR at 600 MHz, ^13^C NMR at 150 MHz, see supplementary materials). The chemical shifts were recorded in parts per million (ppm, δ) relative to residual CHCl_3_ (δ 7.26) for ^1^H NMR and CDCl_3_ (δ 77.00) for ^13^C NMR. The ^1^H NMR splitting patterns were designated as singlet (s), doublet (d), triplet (t), quartet (q), doublet of doublets (dd), and multiplets. All first-order splitting patterns were assigned based on the appearance of the multiplet. Splitting patterns that cannot be easily interpreted were designated multiplet (m). All assignments were confirmed with the aid of two-dimensional experiments (COSY, HSQC, and HMBC; see the SI file). Processing of the spectra was performed using MestReNova software [50]. High-resolution mass spectra were obtained by the electrospray ionization (ESI) technique using an Agilent 6230 TOF LC/MS mass spectrometer and a Synapt G2-Si (Waters) TOF mass spectrometer. IR spectra were recorded using an ATR interface on an Agilent Cary 630 FTIR spectrometer (4000–600 cm^−1^). Optical rotations were measured at 24 °C in an Anton Paar MCP-500 polarimeter. Column chromatography was performed in a Teledyne Isco Combiflash apparatus and analytical thin-layer chromatography (TLC) on aluminum plates precoated with Silica Gel 60F-254.

### 3.2. General Procedures for the Synthesis of Pyrroles

#### 3.2.1. Conventional Heating Methodology

An amount of 0.20 mmol of **2** or **7** was dissolved in 15 mL of toluene, then 3.4 mg (0.02 mmol) of *p*-TsOH acid and (4.00 mmol) of the corresponding primary amine (**a**–**c**) were added. The reaction mixture was refluxed for 5 h and subsequently cooled down to room temperature. The toluene was evaporated, the organic phase was redissolved in AcOEt (30 mL) and then treated with 5% diluted HCl solution (1 × 30 mL), saturated NaHCO_3_ solution (2 × 30 mL), washed with brine (2 × 30 mL), and distilled water (1 × 30 mL). Finally, it was dried over anhydrous Na_2_SO_4_, filtered, and concentrated under reduced pressure.

#### 3.2.2. Microwave Methodology

An amount of 0.04 mmol of **2** or **7** was dissolved in 2 mL of toluene in a 10 mL SiC microwave vial. Next, 0.70 mg (0.004 mmol) of *p*-TsOH acid and (0.8 mmol) of the corresponding primary amine (**a**–**c**) were added. The reaction was heated at 200 °C for 80 min at two 40 min intervals under stirring at 600 rpm in a microwave reactor. The pressure reached under these conditions was 6.8–8.2 bar at 8 W. The toluene was evaporated, and the organic phase redissolved in AcOEt 10 mL, washed with saturated NaCl solution (2 × 10 mL), dried over anhydrous Na_2_SO_4_, and concentrated under reduced pressure.

### 3.3. (25R)-N-benzylpyrrolo[2’,3’,4’,5’:16,17,20,22]cholest-5-ene-3β,26-diyl diacetate (**3a**)

Compound **3a** was purified with a gradient of hexane/ethyl acetate from 10:0 to 9:1. It was obtained as a deep yellow syrup with 80% yield under reflux and 89% yield using MW: [α]_D_ −50.6° (*c* 0.9, CHCl_3_). IR: 2945 (C-H, aliphatic), 1732 (C=O acetates), 1243 (C-N). ^1^H NMR (600 MHz, CDCl_3_) δ: 7.26 (2H, m, *meta*), 7.20 (1H, m, *para*), 6.91 (2H, m, *ortho*), 5.36 (1H, d, *J* = 4.8 Hz, H-6), 4.90 (2H, s, CH_2_-benzyl), 4.59 (1H, m, H-3), 3.82 (1H, dd, *J* = 10.8, 5.9 Hz, H-26a), 3.77 (1H, dd, *J* = 10.8, 6.6 Hz, H-26b), 2.41 (2H, m, CH_2_-15), 2.36 (1H, m, H-23a), 2.32 (2H, m, CH_2_-4), 2.16 (1H, m, H-1a), 2.09 (1H, m, H-23b), 2.02 (3H, s, CH_3_COO-3), 1.98 (3H, s, CH_3_-21), 1.97 (3H, s, CH_3_COO-26), 1.07 (3H, s, CH_3_-19), 0.91 (3H, s, CH_3_-18), 0.84 (3H, d, *J* = 6.7 Hz, CH_3_-27). ^13^C NMR (150 MHz, CDCl_3_) δ: 36.1 (C-1), 27.8 (C-2), 73.9 (C-3), 38.2 (C-4), 140.0 (C-5), 122.3 (C-6), 31.7 (C-7), 30.5 (C-8), 50.8 (C-9), 36.9 (C-10), 20.7 (C-11), 36.9 (C-12), 42.0 (C-13), 60.9 (C-14), 22.2 (C-15), 129.7 (C-16), 135.0 (C-17), 18.5 (C-18), 19.3 (C-19), 109.3 (C-20), 10.0 (C-21), 134.2 (C-22), 26.1 (C-23), 34.2 (C-24), 32.4 (C-25), 69.0 (C-26), 16.7 (C-27), 171.2 (CH_3_COO-3), 170.5 (CH_3_COO-26), 21.4 (CH_3_COO-3), 20.9 (CH_3_COO-26), 139.3 (C-ipso), 128.6 (C-*meta*), 127.0 (C-*para*), 126.0 (C-*ortho*), 48.4 (CH_2_-*benzyl*). HRMS [M + H]^+^ *m*/*z*: Calcd: 586.3896 for C_38_H_52_NO_4_. Found: 586.3905.

### 3.4. (25R)-N-(2-hydroxy)ethylpyrrolo[2’,3’,4’,5’:16,17,20,22]cholest-5-ene-3β,26-diyl diacetate (**3b**)

Compound **3b** was purified with a gradient of hexane/ethyl acetate from 10:0 to 9:1. It was obtained as a deep yellow syrup with 47% yield under reflux and 60% yield using MW: [α]_D_ −48.3° (*c* 0.6, CHCl_3_). IR: 3348 (O-H), 2933 (C-H, aliphatic), 1733 (C=O acetates), 1240 (C-N). ^1^H NMR (600 MHz, CDCl_3_) δ: 5.39 (1H, d, *J* = 5.2 Hz, H-6), 4.60 (1H, m, H-3), 3.94 (2H, m, CH_2_-26), 3.82 (2H, dd, *J* = 6.9, 5.4 Hz, CH_2_-1′), 3.75 (2H, dd, *J* = 5.3, 6.9 Hz, CH_2_-2′), 2.49 (2H, m, CH_2_-15), 2.49 (1H, m, H-23a), 2.31 (2H, m, CH_2_-4), 2.22 (1H, dd, *J* = 13.5, 11.0, H-23b), 2.14 (1H, m, H-1a), 2.05 (3H, s, CH_3_COO-3), 2.02 (3H, s, CH_3_COO-26), 1.95 (3H, s, CH_3_-21), 1.07 (3H, s, CH_3_-19), 0.98 (3H, d, *J* = 6.8 Hz, CH_3_-27), 0.87 (3H, s, CH_3_-18). ^13^C NMR (150 MHz, CDCl_3_) δ: 35.9 (C-1), 27.7 (C-2), 73.9 (C-3), 38.1 (C-4), 140.0 (C-5), 122.3 (C-6), 31.7 (C-7), 30.4 (C-8), 50.7 (C-9), 36.9 (C-10), 20.6 (C-11), 36.8 (C-12), 41.8 (C-13), 60.8 (C-14), 21.9 (C-15), 129.8 (C-16), 135.2 (C-17), 18.3 (C-18), 19.2 (C-19), 109.1 (C-20), 9.8 (C-21), 133.8 (C-22), 26.4 (C-23), 34.3 (C-24), 32.6 (C-25), 68.9 (C-26), 16.8 (C-27), 171.3 (CH_3_COO-3), 170.5 (CH_3_COO-26), 21.4 (CH_3_COO-3), 20.9 (CH_3_COO-26), 47.1 (C-1′), 62.5 (C-2′). HRMS [M + H]^+^ *m*/*z*: Calcd: 540.3689 for C_33_H_50_NO_5_. Found: 540.3699.

### 3.5. (25R)-N-(3-hydroxy)propylpyrrolo[2’,3’,4’,5’:16,17,20,22]cholest-5-ene-3β,26-diyl diacetate (**3c**)

Compound **3c** was purified with a gradient of hexane/ethyl acetate from 10:0 to 9:1. It was obtained as a deep yellow syrup with 76% yield under reflux and 91% yield using MW: [α]_D_ −53.9° (*c* 0.9, CHCl_3_). IR: 3340 (O-H), 2940 (C-H, aliphatic), 1732 (C=O acetates), 1239 (C-N). ^1^H NMR (600 MHz, CDCl_3_) δ: 5.40 (1H, d, *J* = 5.2 Hz, H-6), 4.60 (1H, m, H-3), 3.98 (1H, dd, *J* = 10.8, 6.0 Hz, H-26a), 3.93 (1H, dd, *J* = 10.8, 6.5 Hz, H-26b), 3.80 (2H, dd, *J* = 7.9, 6.5 Hz, CH_2_-1′), 3.64 (2H, dd, *J* = 5.9, 7.9 Hz, CH_2_-3′), 2.50 (2H, m, CH_2_-15), 2.48 (1H, m, H-23a), 2.34 (2H, m, CH_2_-4), 2.21 (1H, dd, *J* = 13.4, 11.0, H-23b), 2.13 (1H, m, H-1a), 2.05 (3H, s, CH_3_COO-3), 2.03 (3H, s, CH_3_COO-26), 1.96 (3H, s, CH_3_-21), 1.08 (3H, s, CH_3_-19), 1.00 (3H, d, *J* = 6.8 Hz, CH_3_-27), 0.88 (3H, s, CH_3_-18). ^13^C NMR (150 MHz, CDCl_3_) δ: 36.0 (C-1), 27.7 (C-2), 73.9 (C-3), 38.1 (C-4), 140.1 (C-5), 122.3 (C-6), 31.8 (C-7), 30.4 (C-8), 50.7 (C-9), 36.9 (C-10), 20.6 (C-11), 36.8 (C-12), 41.9 (C-13), 60.8 (C-14), 22.0 (C-15), 129.3 (C-16), 135.0 (C-17), 18.3 (C-18), 19.2 (C-19), 108.8 (C-20), 9.9 (C-21), 133.3 (C-22), 26.4 (C-23), 34.1 (C-24), 32.6 (C-25), 69.0 (C-26), 16.8 (C-27), 171.4 (CH_3_COO-3), 170.6 (CH_3_COO-26), 21.4 (CH_3_COO-3), 20.9 (CH_3_COO-26), 41.7 (C-1′), 34.4 (C-2′), 60.2 (C-3′). HRMS [M + H]^+^ *m*/*z*: Calcd: 554.3845 for C_34_H_52_NO_5_. Found: 554.3852.

### 3.6. (25R)-22,25-dioxo-27-norcholest-5-en-3β,16-diyl diacetate (**7**)

Compound **7** was purified with a gradient of hexane/ethyl acetate from 10:0 to 7:3. It was obtained as a yellowish powder with a 42% yield under reflux and 63% yield using MW: [α]_D_ +20.9° (*c* 0.1, CHCl_3_). IR: 2936 (C-H, aliphatic), 1726 (C=O ketones), 1725 (C=O acetates), 1240 (C=C). ^1^H NMR (600 MHz, CDCl_3_) δ: 5.34 (1H, d, *J* = 4.7 Hz, H-6), 4.91 (1H, m, H-16), 4.58 (1H, m, H-3), 2.97 (1H, m, H-20), 2.92 (1H, m, H-23a), 2.64 (1H, m, H-24a), 2.60 (1H, m, H-24b), 2.54 (1H, m, H-23b), 2.39 (1H, m, H-15a), 2.29 (2H, m, CH_2_-4), 2.17 (3H, m, CH_3_-26), 2.01 (3H, s, CH_3_COO-3), 1.99 (3H, s, CH_3_COO-16), 1.15 (3H, d, *J* = 7.0 Hz, CH_3_-21), 1.01 (3H, s, CH_3_-19), 0.86 (3H, s, CH_3_-18). ^13^C NMR (150 MHz, CDCl_3_) δ: 36.8 (C-1), 27.7 (C-2), 73.8 (C-3), 38.0 (C-4), 139.6 (C-5), 122.2 (C-6), 31.6 (C-7), 31.2 (C-8), 49.7 (C-9), 36.5 (C-10), 20.7 (C-11), 39.6 (C-12), 41.9 (C-13), 53.9 (C-14), 34.8 (C-15), 75.5 (C-16), 55.1 (C-17), 13.1 (C-18), 19.2 (C-19), 43.1 (C-20), 16.7 (C-21), 211.6 (C-22), 34.6 (C-23), 36.3 (C-24), 207.1 (C-25), 30.0 (C-26), 170.5 (CH_3_COO-3), 170.0 (CH_3_COO-16), 21.4 (CH_3_COO-3), 21.2 (CH_3_COO-16). HRMS (ESI-TOF) *m*/*z* [M + Na]^+^ calcd for 523.3036 for C_30_H_44_NaO_6_, found: 523.3041.

### 3.7. N-Benzylpyrrolo [2′,3′,4′,5′:22,23,24,25]-27-Norcholest-5-ene 3β,16-Diyl Diacetate (**8a**)

Compound **8a** was purified with a gradient of hexane/ethyl acetate from 10:0 to 9:1. It was obtained as a deep yellow syrup in a 50% yield under reflux and a 63% yield using MW: [α]_D_ −6.67° (*c* 0.1, CHCl_3_). IR: 2929 (C-H, aliphatic), 1726 (C=O acetates), 1374 (C=C), 1238 (C-N). ^1^H NMR (600 MHz, CDCl_3_) δ: 7.29 (2H, m, *meta*), 7.22 (1H, m, *para*), 6.88 (2H, m, *ortho*), 5.85 (2H, dd, *J* = 6.6, 3.6, H-23 and H-24), 5.36 (1H, d, *J* = 6.6 Hz, H-6), 5.06 (2H, dd, *J* = 16.8, 59.4, CH_2_-benzyl), 4.97 (1H, m, H-16), 4.60 (1H, m, H-3), 2.99 (1H, m, H-20), 2.31 (3H, m, CH_2_-4, H-15a), 2.09 (3H, m, CH_3_-26), 2.04 (3H, s, CH_3_COO-3), 2.03 (3H, s, CH_3_COO-16), 1.02 (3H, s, CH_3_-19), 0.90 (3H, s, CH_3_-18), 0.84 (3H, d, *J* = 7.2 Hz, CH_3_-21). ^13^C NMR (150 MHz, CDCl_3_) δ: 36.9 (C-1), 27.7 (C-2), 73.8 (C-3), 38.0 (C-4), 139.7 (C-5), 122.3 (C-6), 31.6 (C-7), 31.4 (C-8), 49.9 (C-9), 36.5 (C-10), 20.7 (C-11), 39.6 (C-12), 42.3 (C-13), 54.4 (C-14), 34.8 (C-15), 76.1 (C-16), 60.4 (C-17), 12.7 (C-18), 19.3 (C-19), 28.2 (C-20), 22.6 (C-21), 138.0 (C-22), 102.8 (C-23), 106.1 (C-24), 126.3 (C-25), 12.4 (C-26), 170.5 (CH_3_COO-3), 169.9 (CH_3_COO-16), 21.4 (CH_3_COO-3), 21.1 (CH_3_COO-16), 139.0 (C-*ipso*), 128.7 (C-*meta*), 127.0 (C-*para*), 125.8 (C-*ortho*), 46.4 (CH_2_-*benzyl*). HRMS (ESI-TOF) *m*/*z* [M + H]^+^ calcd for 572.3740 for C_37_H_50_NO_4_, found: 572.3738.

### 3.8. N-(2-hydroxy)ethylpyrrolo[2’,3’,4’,5’:22,23,24,25]-27-norcholest-5-ene 3β,16-diyl diacetate (**8b**)

Compound **8b** was purified with a gradient of hexane/ethyl acetate from 10:0 to 7:3. It was obtained as a brown syrup in a 20% yield under reflux and a 31% yield using MW: [α]_D_ +13.5° (*c* 0.1, CHCl_3_). IR: 3342 (O-H), 2939 (C-H, aliphatic), 1721 (C=O acetates), 1375 (C=C), 1236 (C-N). ^1^H NMR (600 MHz, CDCl_3_) δ: 5.78 (2H, dd, *J* = 7.6, 3.6, H-23 and H-24), 5.36 (1H, d, *J* = 4.7 Hz, H-6), 4.98 (1H, m, H-16), 4.60 (1H, m, H-3), 4.04 (1H, m, H-1′a), 3.91 (1H, m, H-1′b), 3.81 (2H, m, CH_2_-2′), 3.06 (1H, m, H-20), 2.37 (1H, m, H-15a), 2.32 (2H, m, CH_2_-4), 2.20 (3H, m, CH_3_-26), 2.03 (3H, s, CH_3_COO-3), 1.84 (3H, s, CH_3_COO-16), 1.22 (3H, d, *J* = 6.6 Hz, CH_3_-21), 1.04 (3H, s, CH_3_-19), 0.95 (3H, s, CH_3_-18). ^13^C NMR (150 MHz, CDCl_3_) δ: 36.9 (C-1), 27.7 (C-2), 73.8 (C-3), 38.0 (C-4), 139.7 (C-5), 122.3 (C-6), 31.6 (C-7), 31.4 (C-8), 49.9 (C-9), 36.5 (C-10), 20.8 (C-11), 39.7 (C-12), 42.4 (C-13), 54.3 (C-14), 35.0 (C-15), 76.5 (C-16), 59.4 (C-17), 12.9 (C-18), 19.3 (C-19), 28.2 (C-20), 23.4 (C-21), 137.7 (C-22), 102.7 (C-23), 106.2 (C-24), 126.5 (C-25), 12.7 (C-26), 170.5 (CH_3_COO-3), 170.0 (CH_3_COO-16), 21.4 (CH_3_COO-3), 21.1 (CH_3_COO-16), 45.2 (C-1′), 62.5 (C-2′). HRMS (ESI-TOF) *m*/*z* [M + H]^+^ calcd for 526.3532 for C_32_H_48_NO_5_, found: 526.3540.

### 3.9. N-(3-hydroxy)propylpyrrolo[2’,3’,4’,5’:22,23,24,25]-27-norcholest-5-ene 3β,16-diyl diacetate (**8c**)

Compound **8c** was purified with a gradient of hexane/ethyl acetate from 10:0 to 7:3. It was obtained as a brown syrup in a 25% yield under reflux and a 41% yield using MW: [α]_D_ +11° (*c* 0.1, CHCl_3_). IR: 3407 (O-H), 2930 (C-H, aliphatic), 1724 (C=O acetates), 1372 (C=C), 1239 (C-N). ^1^H NMR (600 MHz, CDCl_3_) δ: 5.75 (2H, s, H-23 and H-24), 5.36 (1H, d, *J* = 4.7 Hz, H-6), 4.95 (1H, m, H-16), 4.59 (1H, m, H-3), 4.00 (1H, m, H-1′a), 3.82 (1H, m, H-1′b), 3.74 (2H, m, CH_2_-3′), 3.05 (1H, m, H-20), 2.35 (1H, m, H-15a), 2.30 (2H, m, CH_2_-4), 2.18 (3H, m, CH_3_-26), 2.03 (3H, s, CH_3_COO-3), 1.82 (3H, s, CH_3_COO-26), 1.23 (3H, d, *J* = 6.5 Hz, CH_3_-21), 1.03 (3H, s, CH_3_-19), 0.90 (3H, s, CH_3_-18). ^13^C NMR (150 MHz, CDCl_3_) δ: 36.9 (C-1), 27.7 (C-2), 73.9 (C-3), 38.0 (C-4), 139.7 (C-5), 122.3 (C-6), 31.6 (C-7), 31.4 (C-8), 49.9 (C-9), 36.5 (C-10), 20.8 (C-11), 39.7 (C-12), 42.4 (C-13), 54.3 (C-14), 34.9 (C-15), 76.2 (C-16), 60.3 (C-17), 12.8 (C-18), 19.3 (C-19), 28.9 (C-20), 23.0 (C-21), 137.3 (C-22), 102.3 (C-23), 105.9 (C-24), 125.6 (C-25), 12.4 (C-26), 170.5 (CH_3_COO-3), 169.9 (CH_3_COO-16), 21.4 (CH_3_COO-3), 21.2 (CH_3_COO-16), 42.4 (C-1′), 34.2 (C-2′), 59.6 (C-3′). HRMS (ESI-TOF) *m*/*z* [M + H]^+^ calcd for 540.3689 for C_33_H_50_NO_5_, found: 540.3690.

### 3.10. General Remarks for Bioassays

The activity of the norcholestane pyrroles **8a**–**c** as acaricides and plant-growth promoters was evaluated under laboratory and greenhouse conditions, respectively. Due to their lack of stability in the solution for longer than one week and impossibility to be preserved for the duration of the studies, compounds **3a**–**c** were not investigated. To achieve these concentrations, stock solutions of **8a**–**c** (5 g/L) were prepared in dimethyl sulfoxide (DMSO). These stock solutions were diluted in distilled water to either 5 mg/L for the acaricide bioassay or 0.05 mg/L for the plant-growth-promoting bioassay. The concentration of DMSO did not exceed 0.1% (*v*/*v*) in the final solutions used for treatments. These concentrations were established based on previous experiences in similar studies and in experiments involving steroidal compounds [51,52,53,54].

### 3.11. Acaricidal Activity in the Two-Spotted Spider Mite (Tetranychus urticae Koch) under Laboratory Conditions

#### 3.11.1. Bioassay for *T. urticae* Adults

The acaricide-immersed leaf technique was used in this bioassay [55]. Leaf discs of habanero pepper (5 cm in diameter) were cut and immersed for 5 s in 250 mL beakers containing different solutions (compounds **8a**–**c**). After immersion, the leaf discs were dried at room temperature for 30 min and then placed adaxial side up on moistened cotton in Petri dishes (9.0 × 1.5 cm). To keep the mites from escaping, wet cotton was placed around the edges of the leaf discs. Fifteen adults of *T. urticae* were transferred to each leaf disc and mortality was recorded after 24 and 48 h. The Petri dishes were kept in the laboratory at 24 ± 2 °C and a photoperiod of 14 h of light and 10 h of darkness. Mites that remained motionless after being touched with a fine brush were assumed to be dead. A Petri dish represented a replicate, and ten replicates were included for each compound.

#### 3.11.2. Bioassay for *T*. *urticae* Eggs

Twenty adult females were transferred to 5 cm diameter habanero pepper leaf discs placed on wet cotton in Petri dishes (9.0 × 1.5 cm). After 24 h, all adults and some eggs were removed, leaving only 20 eggs per leaf disc. The leaf discs containing the eggs were immersed for 5 s in the solutions and placed back in the Petri dishes. After six days, the mortality of the eggs was recorded. Eggs with dark coloration and those that did not hatch were considered dead. A Petri dish represented a replicate, and eight replicates were included for each compound.

### 3.12. Plant Growth Promotion in Habanero Pepper Plants (Capsicum chinense Jacq) under Greenhouse Conditions

#### 3.12.1. Establishment of Potted Plants

The study was established under greenhouse conditions (25–35 °C, R.H. 55–75%). The vegetative material used was habanero pepper (*Capsicum chinense* Jacq) of the Prime variety. Twenty days after emergence, the plants were transplanted into 2 L pots filled with local soil. The plants in pots were kept under constant irrigation to achieve field capacity, and triple 17 fertilizer was added for nutrition at a rate of 2 g/L every second day.

#### 3.12.2. Compound Application and Evaluation

At 10 and 17 days after transplanting into pots, with the aid of a hand sprayer, compounds **8a**–**c** were applied directly to the foliage of the plants until the drip point. Distilled water was used as the negative control. Each plant represented one replicate, and 7–15 plants were used for each compound. The variables evaluated were plant height (measured in cm from the base of the stem to the terminal apex) and the number of leaves. Whole plants were taken and sectioned into leaves, stems, and roots to evaluate dry biomass.

### 3.13. Data Analysis

For data analysis, a completely randomized experimental design was set. Analysis of variance and comparison of means by Tukey’s method was applied and differences between means were considered significant if *p* < 0.05.

## 4. Conclusions

We successfully synthesized fused pyrroles in the side chain of cholestane and norcholestane skeletons. For the cholestane pyrroles, we revisited the Paal–Knorr reaction in kryptogenin 3,26-diacetate using three primary amines: ethanolamine, propanolamine, and benzylamine. We developed two mild methodologies employing conventional reflux and MW-assisted heating. The best solvent for the Paal–Knorr reactions was toluene, so SiC vials were used to conduct these reactions to undergo heating. In addition, all the reactions were carried out using a catalytic amount of *p*-TsOH. The norcholestane skeleton was constructed from diosgenin, with a series of reactions in the side chain. Those reactions involve the opening of the side chain and the oxidation of the primary hydroxyl group at C-26 to obtain an aldehyde that constitutes a 1,5-dicarbonyl system. The latter was transformed into the required 1,4-dicarbonyl system through a C-C cleavage of the aldehyde using ethanol and 3,4,5-trimethoxyaniline. Finally, the Paal–Knorr conditions developed for kryptogenin pyrroles were also applied, successfully providing trisubstituted pyrroles. These norcholestane pyrroles **8a**–**c** were evaluated as acaricides against adults and eggs of the two-spotted spider mite (*T. urticae*). For adults, **8a** and **8b** caused significant mortality at 24 h after spraying. At 48 and 72 h after spraying, all compounds caused significant mortality, and at 72 h, **8c** produced the highest effect. For eggs, **8b** and **8c** caused significant mortality. It is important to note that all compounds had a modest effect on adults, and **8c** showed a highly lethal effect on eggs. The same compounds were tested as plant growth promoters in habanero pepper (*C. chinense*). Treatments had no meaningful effect on plant growth (plant height and number of leaves per plant). However, regarding biomass accumulation, root dry biomass was significantly higher in plants treated with **8c**. Therefore, **8c** could exert a critical acaricidal effect and, simultaneously, a growth-promoting effect on roots, thereby providing a twofold benefit to the treated plants. Therefore, we believe these compounds are promising candidates for further optimization, such as improved bioavailability by enhancing their polarity or boosting their solubility in aqueous media.

## Figures and Tables

**Table 1:** Effect of different solvents on synthesizing the fused cholestane pyrrole **3a** at reflux.

Entry	Solvent ^[a]^	Reaction Time (h) ^[b]^	Yield of 3a (%) ^[c]^
1	acetonitrile	6	38
2	ethanol	6	45
3	toluene	5	80
4	xylene	5	65
5	DMF	6	32

^[a]^ All the reactions were conducted at reflux, except for DMF where the temperature was kept controlled at 150 °C, as at higher temperatures, multiple degradation products were observed. ^[b]^ Maximum yield occurred within these reaction times. ^[c]^ Isolated yields.

**Table 2:** Selected ^1^H NMR chemical shifts (in ppm) for pyrroles **3a**–**c** and **8a**–**c**.

Position	3a	3b	3c	8a	8b	8c
3	4.59	4.60	4.60	4.60	4.60	4.59
6	5.36	5.39	5.40	5.36	5.36	5.36
16	-	-	-	4.97	4.98	4.95
18	0.91	0.87	0.88	0.90	0.95	0.90
19	1.07	1.07	1.08	1.02	1.04	1.03
20	-	-	-	2.99	3.06	3.05
21	1.98	1.95	1.96	0.84	1.22	1.23
23, 24	-	-	-	5.85	5.78	5.75
26	3.82, 3.77	3.94	3.98, 3.93	2.09	2.20	2.18
27	0.84	0.98	1.00	-	-	-

All NMR spectra were acquired in CDCl_3_ from a 600 MHz NMR spectrometer.

**Table 3:** Selected ^13^C NMR chemical shifts (in ppm) for pyrroles **3a**–**c** and **8a**–**c**.

Position	3a	3b	3c	8a	8b	8c
15	22.2	21.9	22.0	34.8	35.0	34.9
16	129.7	129.8	129.3	76.1	76.5	76.2
17	135.0	135.2	135.0	60.4	59.4	60.3
18	18.5	18.3	18.3	12.7	12.9	12.8
19	19.3	19.2	19.2	19.3	19.3	19.3
20	109.3	109.1	108.8	28.2	28.2	28.9
21	10.0	9.8	9.9	22.6	23.4	23.0
22	134.2	133.8	133.3	138.0	137.7	137.3
23	26.1	26.4	26.4	102.8	102.7	102.3
24	34.2	34.3	34.1	106.1	106.2	105.9
25	32.4	32.6	32.6	126.3	126.5	125.6
26	69.0	68.9	69.0	12.4	12.7	12.4
27	16.7	16.8	16.8	-	-	-

All NMR spectra were acquired in CDCl_3_ from a 600 MHz NMR spectrometer.

**Table 4:** Means ± standard error of the mortality of adults and eggs of *T. urticae* treated with compounds **8a**–**c**.

Compound	% Mortality of Adults			
	24 h	48 h	72 h	% Mortality of Eggs
**8a**	11.3 ± 2.4	16.6 ± 1.1	31.3 ± 2.6	26.25 ± 3.9
**8b**	9.3 ± 1.4	19.3 ± 3.0	26.0 ± 2.3	48.75 ± 4.7
**8c**	5.3 ± 1.6	17.3 ± 1.7	39.3 ± 2.7	56.87 ± 3.12
Control	2.0 ± 1.0	11.3 ± 2.2	23.3 ± 2.4	14.37 ± 5.38

**Table 5:** Means ± standard error of growth and biomass accumulation in habanero pepper plants treated with foliar applications of compounds **8a**–**c**. The applications were conducted 10 and 17 days after transplanting, and the evaluations were performed 25 days after transplanting.

Compound	Plant Height (cm)	Number of Leaves/Plant	Stem Dry Biomass (g)	Leaves Dry Biomass (g)	Root Dry Biomass (g)
**8a**	13.5 ± 0.51	9.0 ± 0.16	0.1 ± 0.00	0.37 ± 0.01	0.10 ± 0.00
**8b**	13.2 ± 0.44	8.6 ± 0.27	0.1 ± 0.00	0.31 ± 0.04	0.12 ± 0.01
**8c**	12.7 ± 0.28	8.6 ± 0.13	0.1 ± 0.00	0.35 ± 0.02	0.15 ± 0.02
Control *	13.2 ± 0.40	8.6 ± 0.18	0.1 ± 0.00	0.34 ± 0.02	0.10 ± 0.00

* The control was Cystar, a cytokinin.

## Supplementary Materials

The following supporting information can be downloaded at: https://www.mdpi.com/article/10.3390/molecules27238466/s1, Figure S1: ^1^H NMR CDCl_3_, 600 MHz compound **3a**; Figure S2: ^13^C NMR CDCl_3_, 150 MHz compound **3a**; Figure S3: COSY experiment compound **3a**; Figure S4: HSQC experiment compound **3a**; Figure S5: HMBC experiment compound **3a**; Figure S6: ^1^H NMR CDCl_3_, 600 MHz compound **3b**; Figure S7: ^13^C NMR CDCl_3_, 150 MHz compound **3b**; Figure S8: COSY experiment compound **3b**; Figure S9: HSQC experiment compound **3b**; Figure S10: HMBC experiment compound **3b**; Figure S11: ^1^H NMR CDCl_3_, 600 MHz compound **3c**; Figure S12: ^13^C NMR CDCl_3_, 150 MHz compound **3c**; Figure S13: COSY experiment compound **3c**; Figure S14: HSQC experiment compound **3c**; Figure S15: HMBC experiment compound **3c**; Figure S16: ^1^H NMR CDCl_3_, 600 MHz compound **7**; Figure S17: ^13^C NMR CDCl_3_, 150 MHz compound **7**; Figure S18: COSY experiment compound **7**; Figure S19: HSQC experiment compound **7**; Figure S20: HMBC experiment compound **7**; Figure S21: ^1^H NMR CDCl_3_, 600 MHz compound **8a**; Figure S22: ^13^C NMR CDCl_3_, 150 MHz compound **8a**; Figure S23: COSY experiment compound **8a**; Figure S24: HSQC experiment compound **8a**; Figure S25: HMBC experiment compound **8a**; Figure S26: ^1^H NMR CDCl_3_, 600 MHz compound **8b**; Figure S27: ^13^C NMR CDCl_3_, 150 MHz compound **8b**; Figure S28: COSY experiment compound **8b**; Figure S29: HSQC experiment compound **8b**; Figure S30: HMBC experiment compound **8b**; Figure S31: ^1^H NMR CDCl_3_, 600 MHz compound **8c**; Figure S32: ^13^C NMR CDCl_3_, 150 MHz compound **8c**; Figure S33: COSY experiment compound **8c**; Figure S34: HSQC experiment compound **8c**; Figure S35: HMBC experiment compound **8c**.

## Sample Availability

Samples of the compounds **3a**–**c** and **8a**–**c** are available from the authors.
