# Supplementary material for: Fused Pyrroles in Cholestane and Norcholestane Side Chains: Acaricidal and Plant Growth-Promoting Effects
Source: Molecules. 2022 Dec 2;27(23):8466. doi: 10.3390/molecules27238466 (PMC9737654; doi:10.3390/molecules27238466)
Supplement: Supplementary file 1 [file molecules-27-08466-s001.zip › molecules-2040024-supplementary.pdf]

*Table of Content*


1. **Figure S1**. $^{1}H$ NMR $CDCl_3$, 600 MHz compound **3a**. S3
2. **Figure S2**. $^{13}C$ NMR $CDCl_3$, 150 MHz compound **3a**. S3
3. **Figure S3**. COSY experiment compound **3a**. S4
4. **Figure S4**. HSQC experiment compound **3a**. S4
5. **Figure S5**. HMBC experiment compound **3a**. S5
6. **Figure S5**. $^{1}H$ NMR $CDCl_3$, 600 MHz compound **3b**. S5
7. **Figure S7**. $^{13}C$ NMR $CDCl_3$, 150 MHz compound **3b**. S6
8. **Figure S8**. COSY experiment compound **3b**. S6
9. **Figure S9**. HSQC experiment compound **3b**. S7
10. **Figure S10**. HMBC experiment compound **3b**. S7
11. **Figure S11**. $^{1}H$ NMR $CDCl_3$, 600 MHz compound **3c**. S8
12. **Figure S12**. $^{13}C$ NMR $CDCl_3$, 150 MHz compound **3c**. S8
13. **Figure S13**. COSY experiment compound **3c**. S9
14. **Figure S14**. HSQC experiment compound **3c**. S9
15. **Figure S15**. HMBC experiment compound **3c**. S10
16. **Figure S16**. $^{1}H$ NMR $CDCl_3$, 600 MHz compound **7**. S10
17. **Figure S17**. $^{13}C$ NMR $CDCl_3$, 150 MHz compound **7**. S11
18. **Figure S18**. COSY experiment compound **7**. S11
19. **Figure S19**. HSQC experiment compound **7**. S12
20. **Figure S20**. HMBC experiment compound **7**. S12
21. **Figure S21**. $^{1}H$ NMR $CDCl_3$, 600 MHz compound **8a**. S13
22. **Figure S22**. $^{13}C$ NMR $CDCl_3$, 150 MHz compound **8a**. S13
23. **Figure S23**. COSY experiment compound **8a**. S14
24. **Figure S24**. HSQC experiment compound **8a**. S14
25. **Figure S25**. HMBC experiment compound **8a**. S15
26. **Figure S26**. $^{1}H$ NMR $CDCl_3$, 600 MHz compound **8b**. S15
27. **Figure S27**. $^{13}C$ NMR $CDCl_3$, 150 MHz compound **8b**. S16
28. **Figure S28**. COSY experiment compound **8b**. S16
29. **Figure S29**. HSQC experiment compound **8b**. S17
30. **Figure S30**. HMBC experiment compound **8b**. S17
31. **Figure S31**. $^{1}H$ NMR $CDCl_3$, 600 MHz compound **8c**. S18
32. **Figure S32**. $^{13}C$ NMR $CDCl_3$, 150 MHz compound **8c**. S18
33. **Figure S33**. COSY experiment compound **8c**. S19
34. **Figure S34**. HSQC experiment compound **8c**. S19
35. **Figure S35**. HMBC experiment compound **8c**. S20

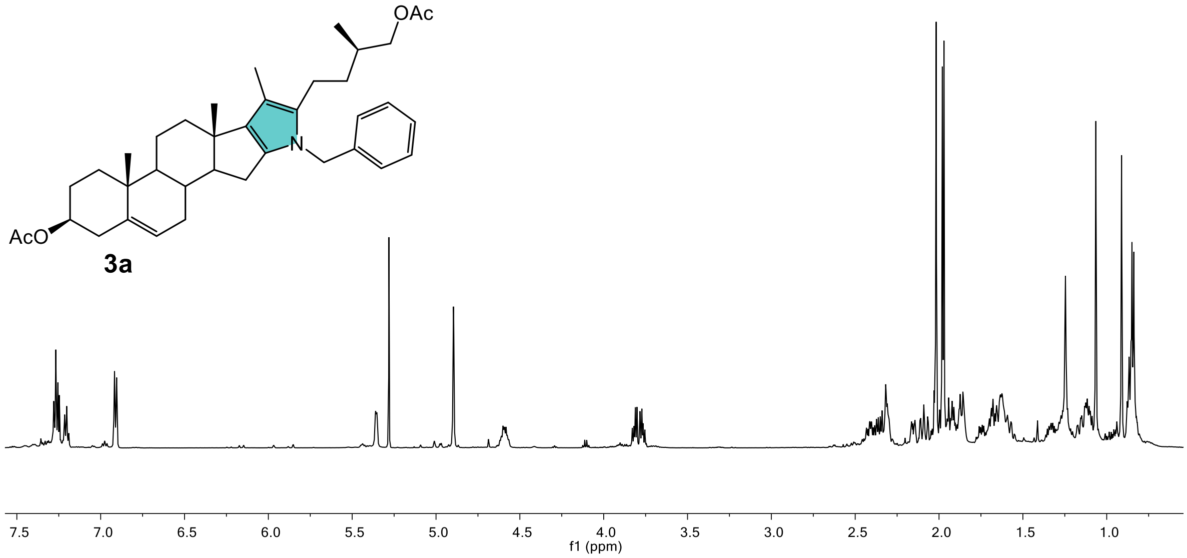


**Figure S1**. $^{1}$H NMR $CDCl_3$, 600 MHz compound **3a**.

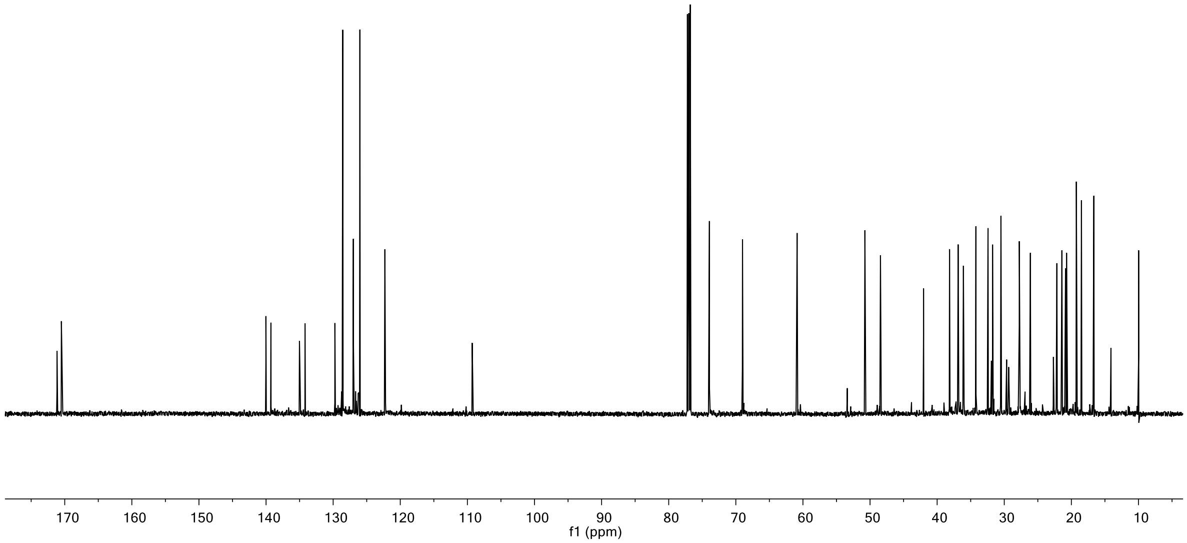


**Figure S2**. $^{13}$C NMR $CDCl_3$, 150 MHz compound **3a**.

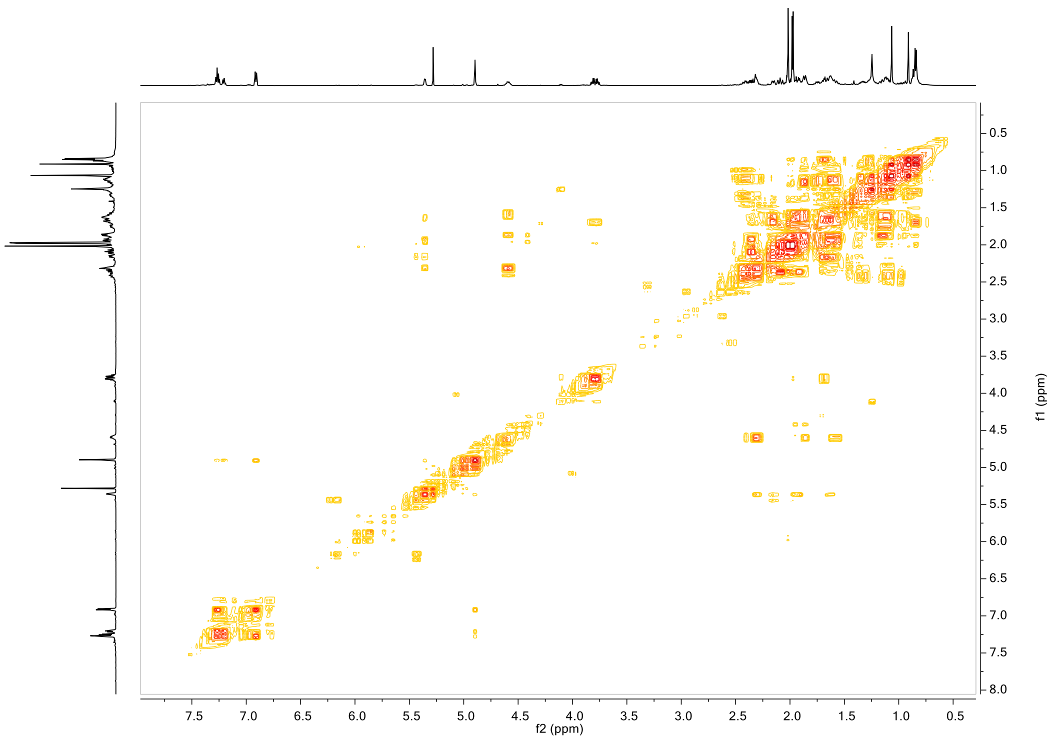


**Figure S3**. COSY experiment compound **3a**.

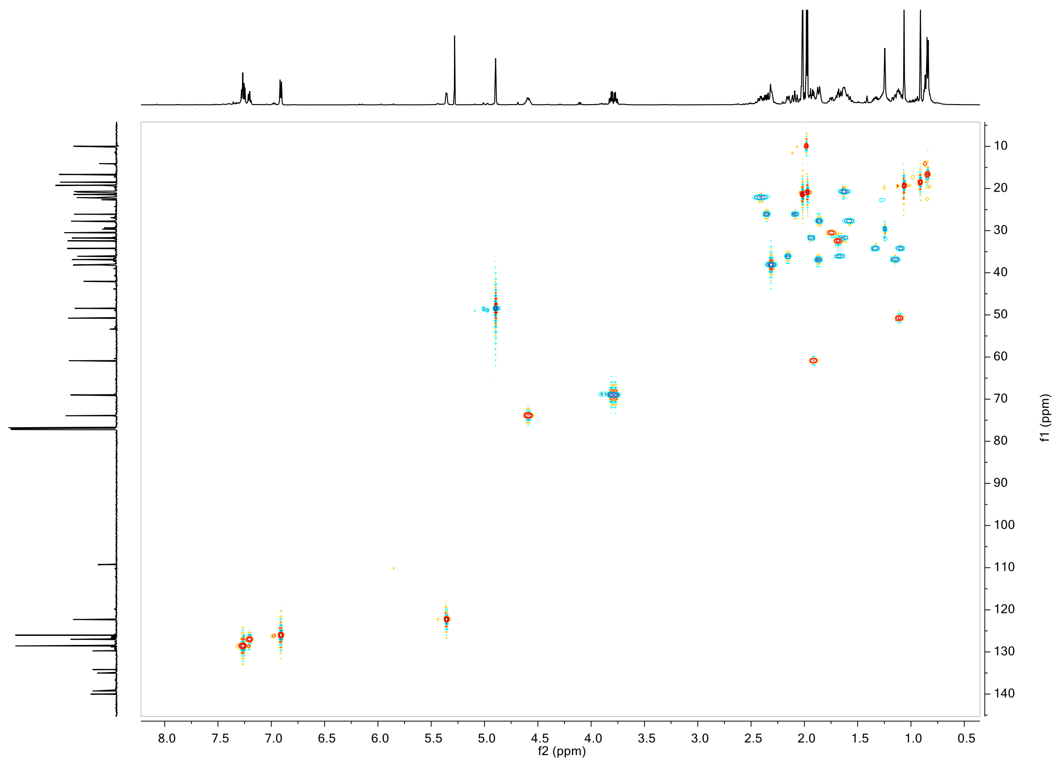


**Figure S4**. HSQC experiment compound **3a**.

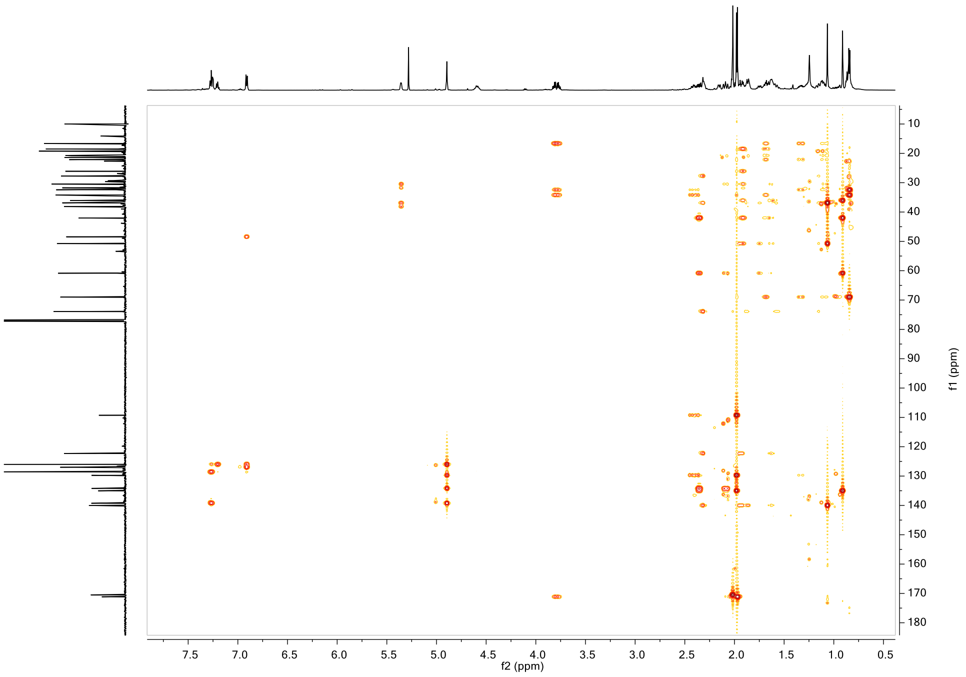


**Figure S5**. HMBC experiment compound **3a**.

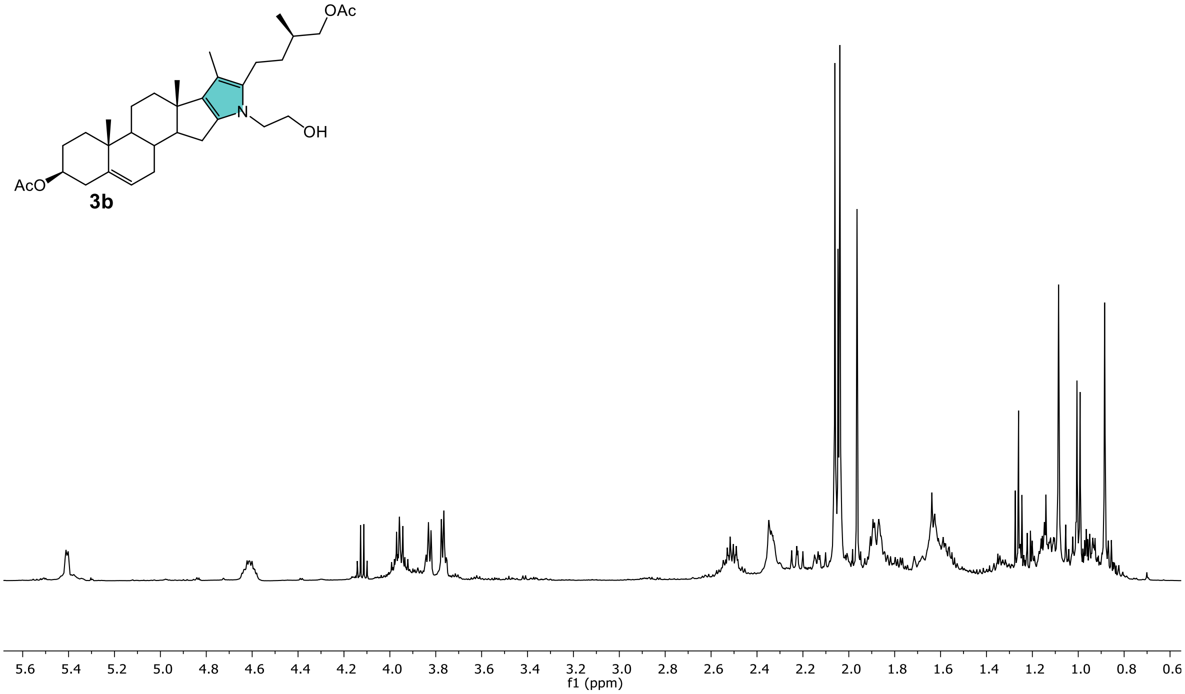


**Figure S6**. $^{1}$H NMR $CDCl_3$, 600 MHz compound **3b**.

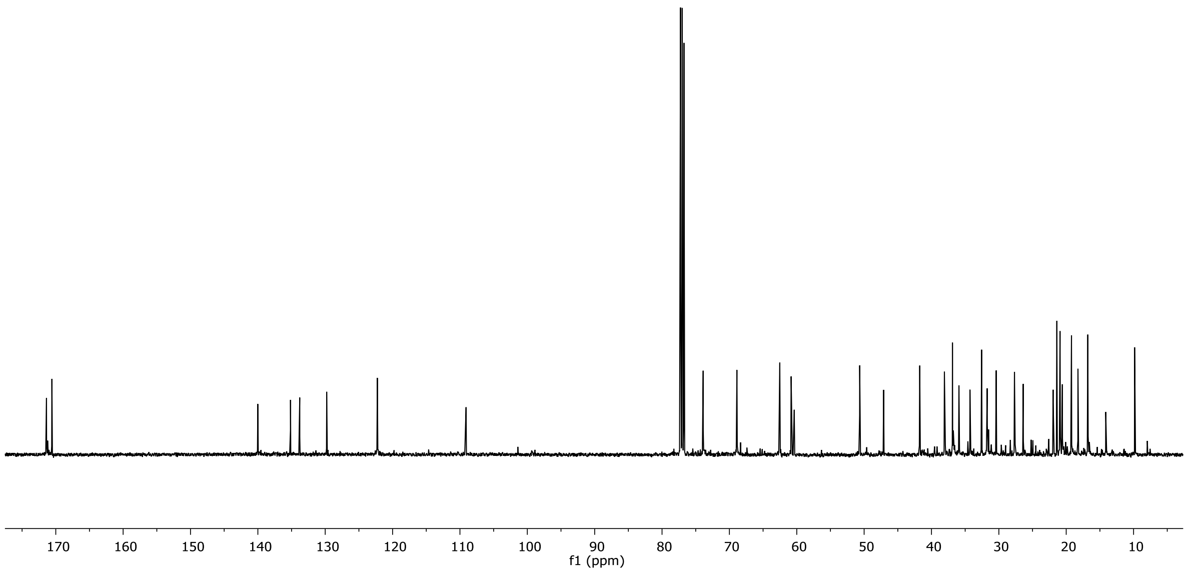


**Figure S7**. $^{13}C$ NMR $CDCl_3$, 150 MHz compound **3b**.

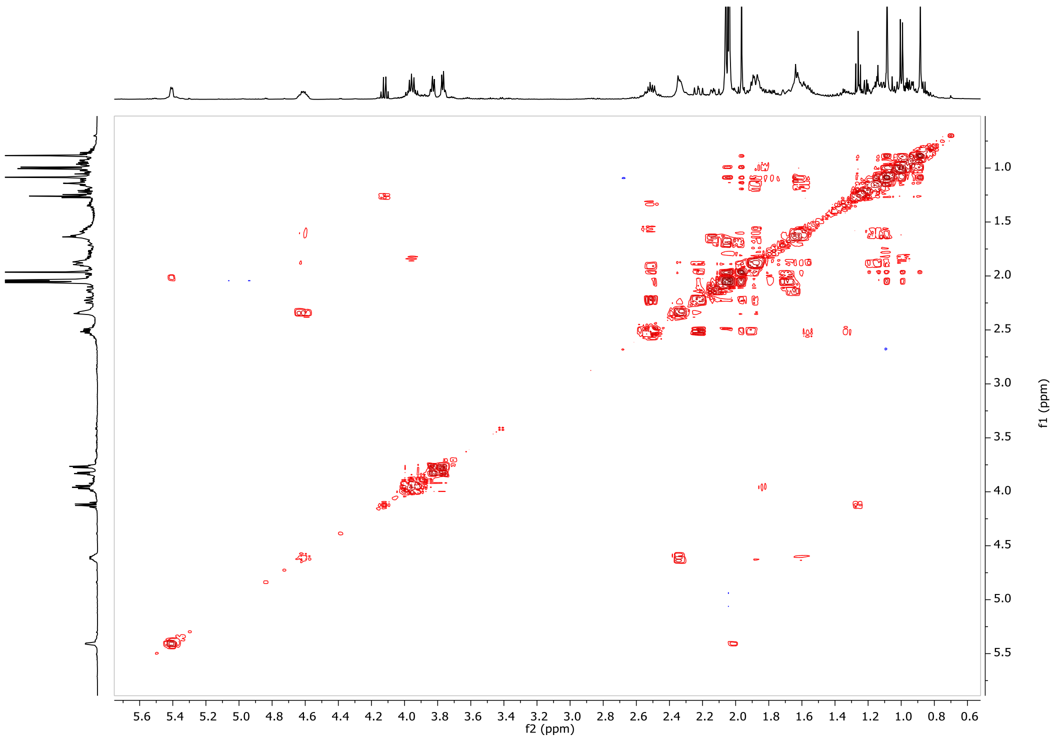


**Figure S8**. COSY experiment compound **3b**.

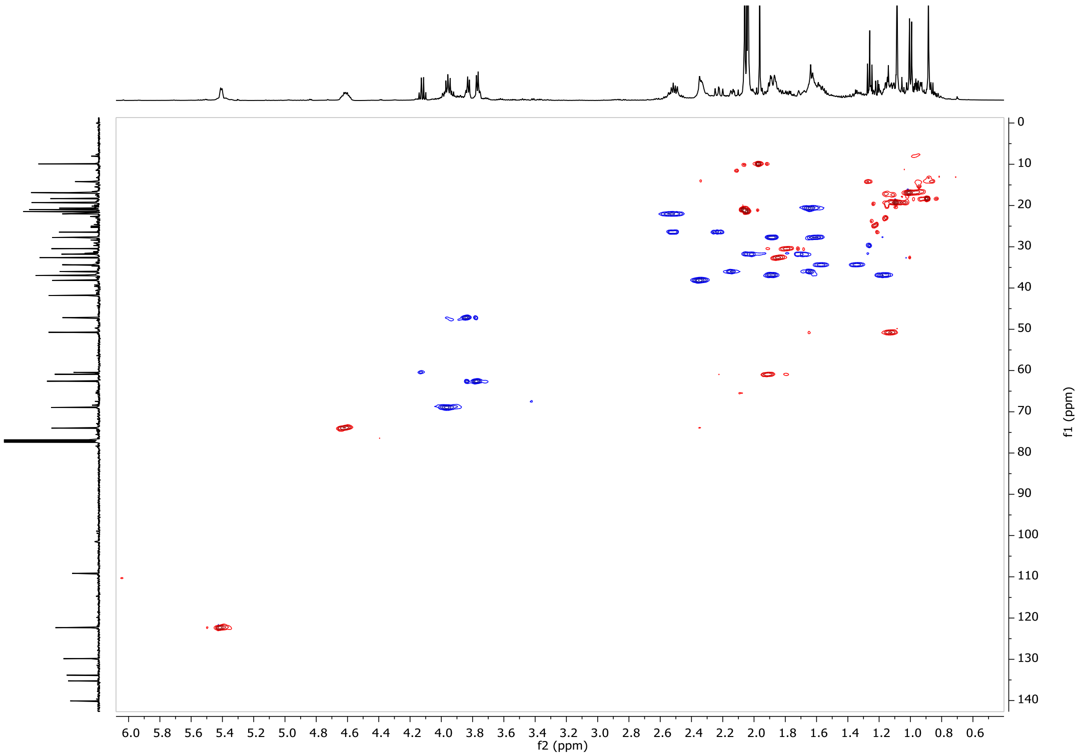


**Figure S9**. HSQC experiment compound **3b**.

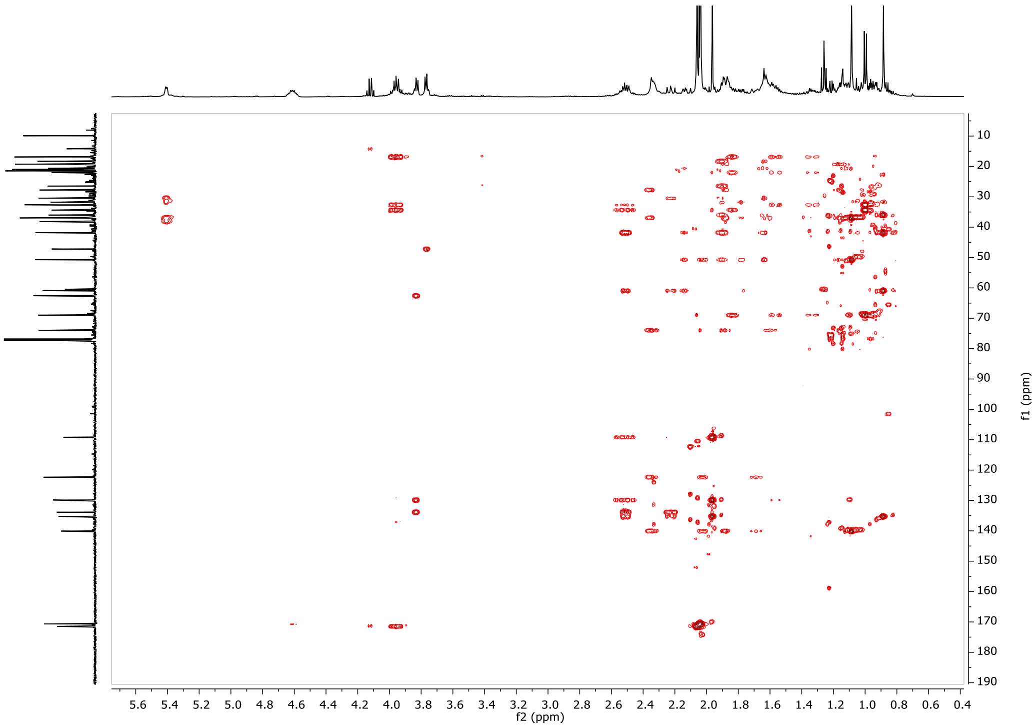


**Figure S10**. HMBC experiment compound **3b**.

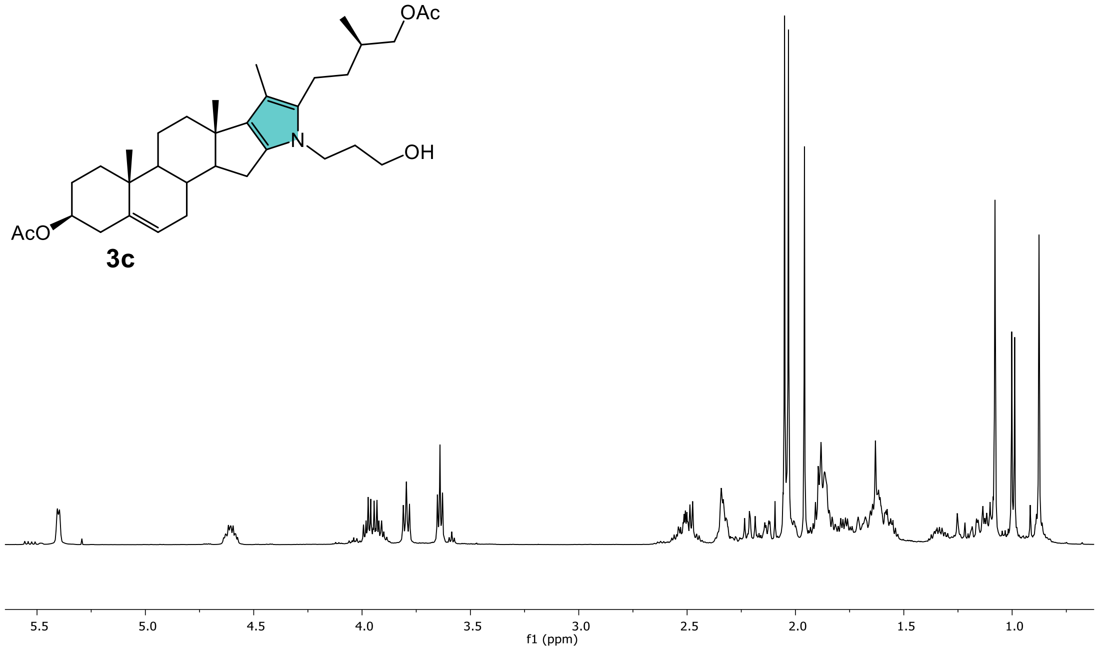


**Figure S11**. $^{1}$H NMR $CDCl_3$, 600 MHz compound **3c**.

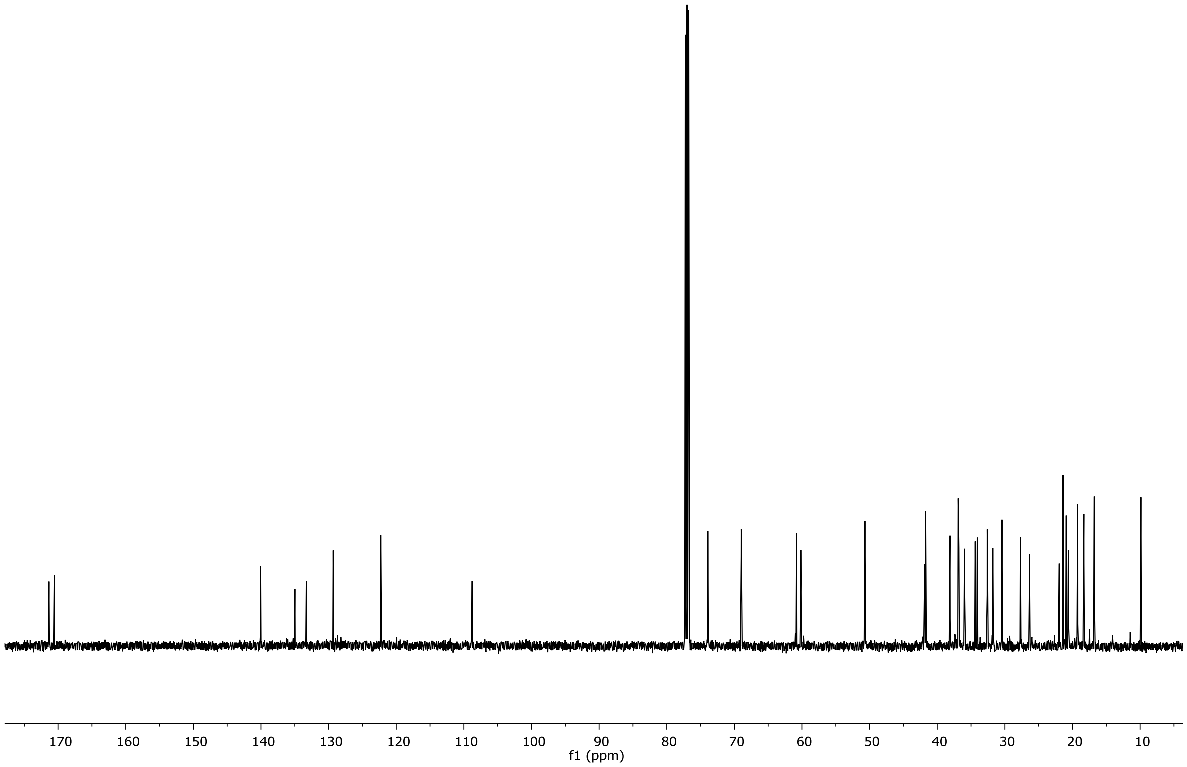


**Figure S12**. $^{13}$C NMR $CDCl_3$, 150 MHz compound **3c**.

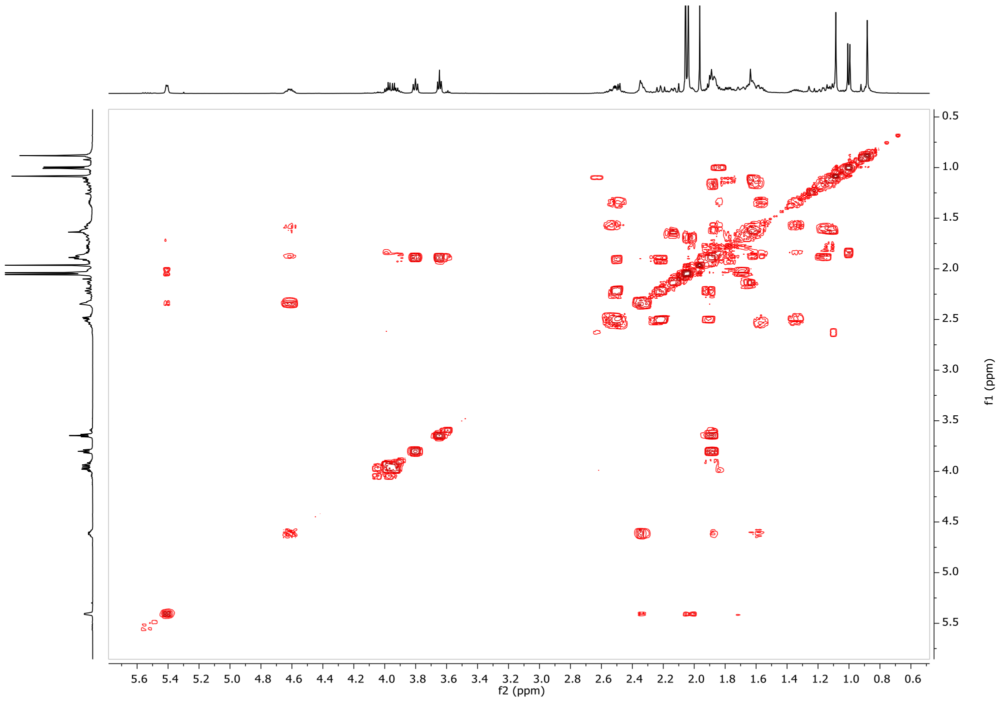


**Figure S13**. COSY experiment compound **3c**.

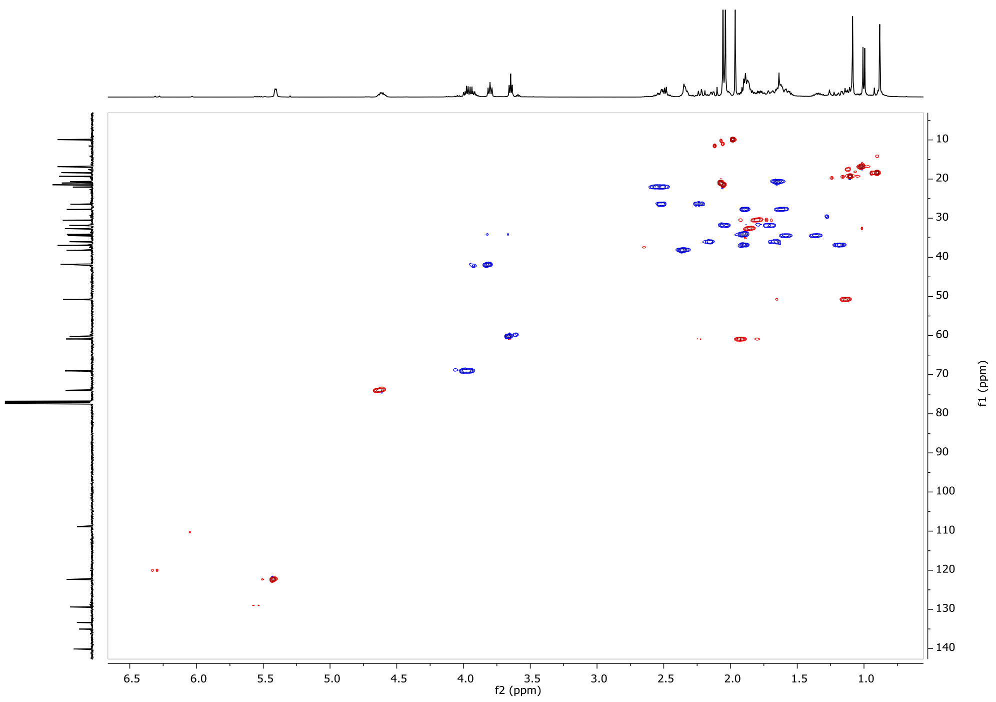


**Figure S14**. HSQC experiment compound **3c**.

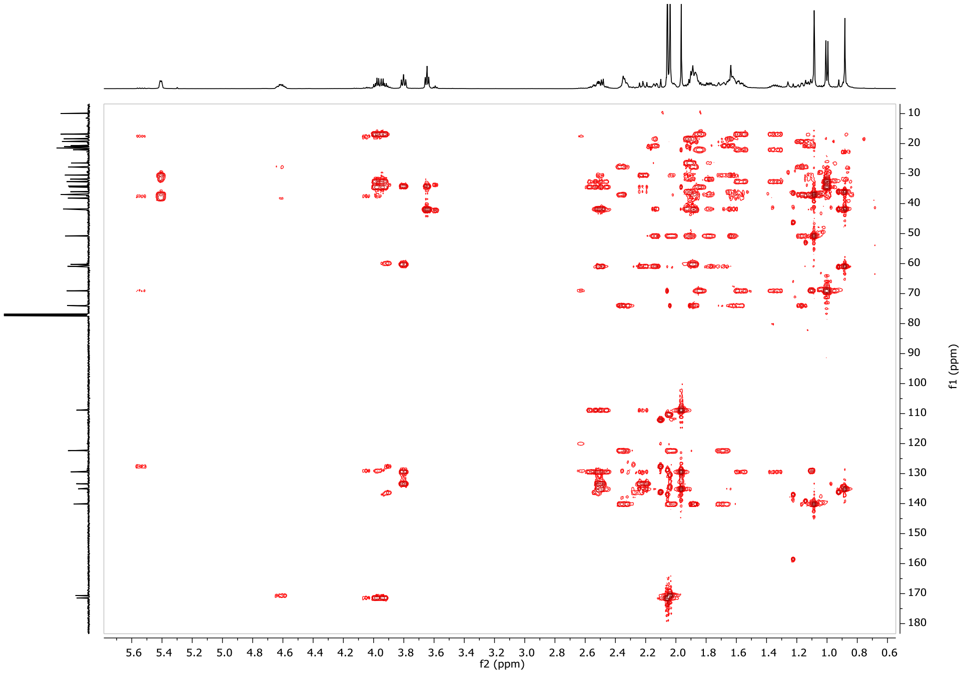


**Figure S15**. HMBC experiment compound **3c**.

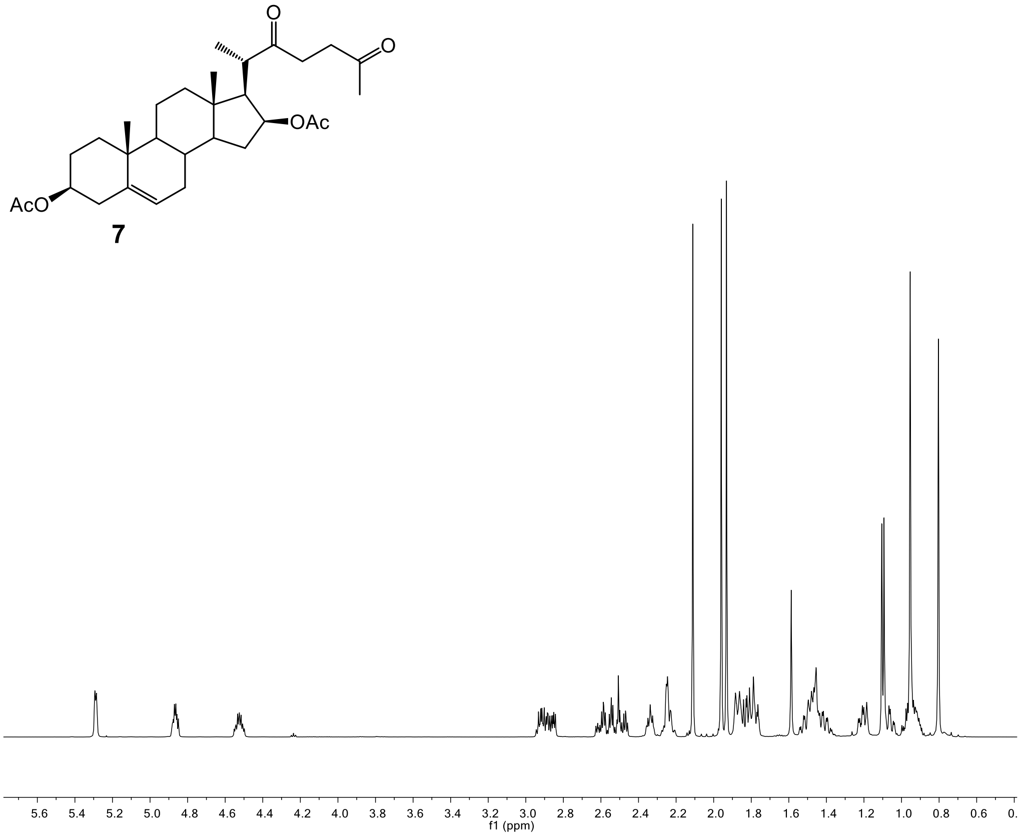


**Figure S16**. $^{1}H$ NMR $CDCl_3$, 600 MHz compound **7**.

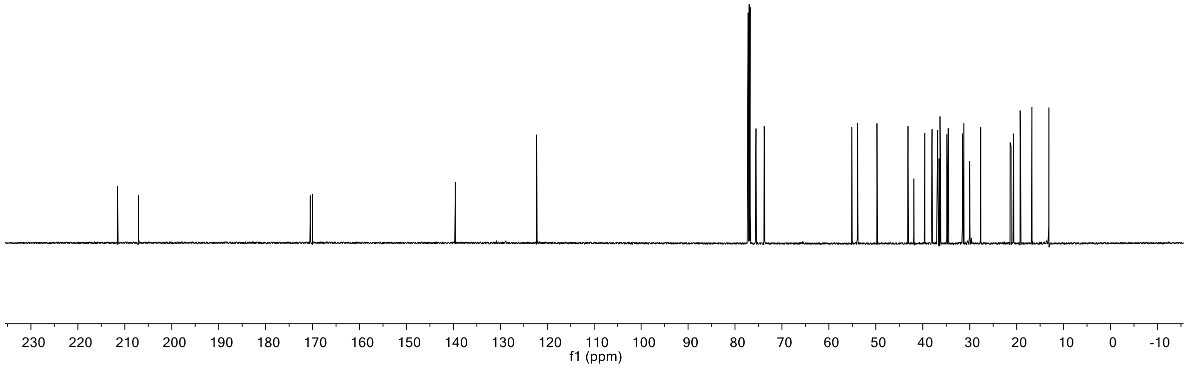


**Figure S17**. $^{13}C$ NMR $CDCl_3$, 150 MHz compound **7**.

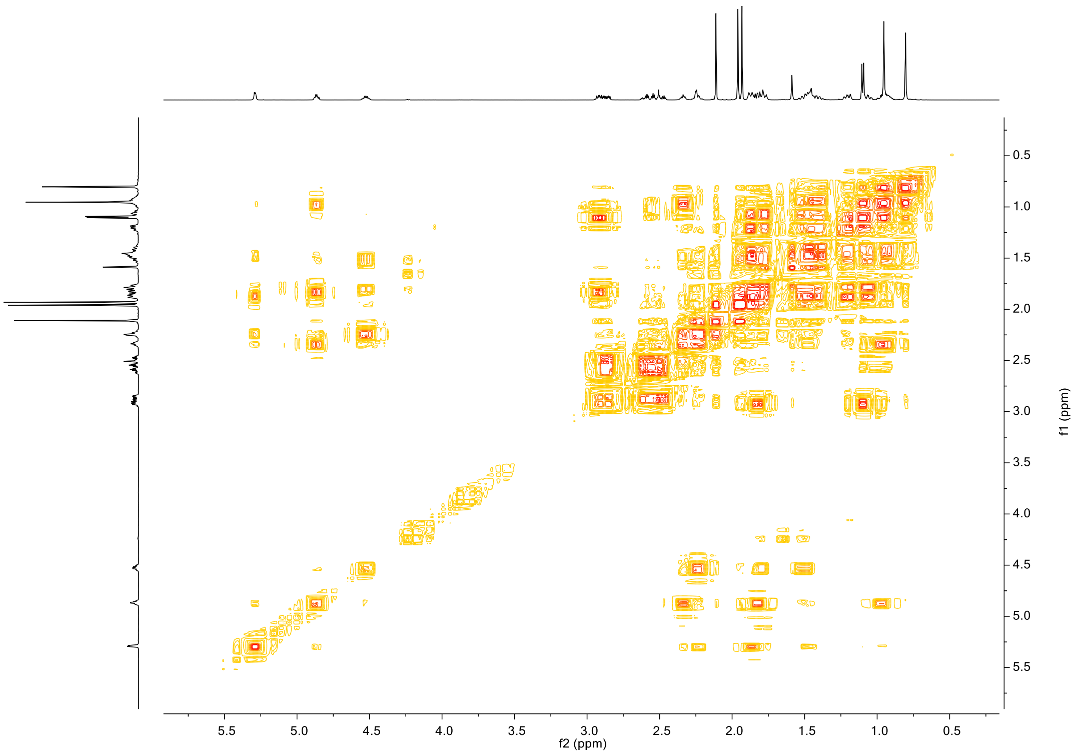


**Figure S18**. COSY experiment compound **7**.

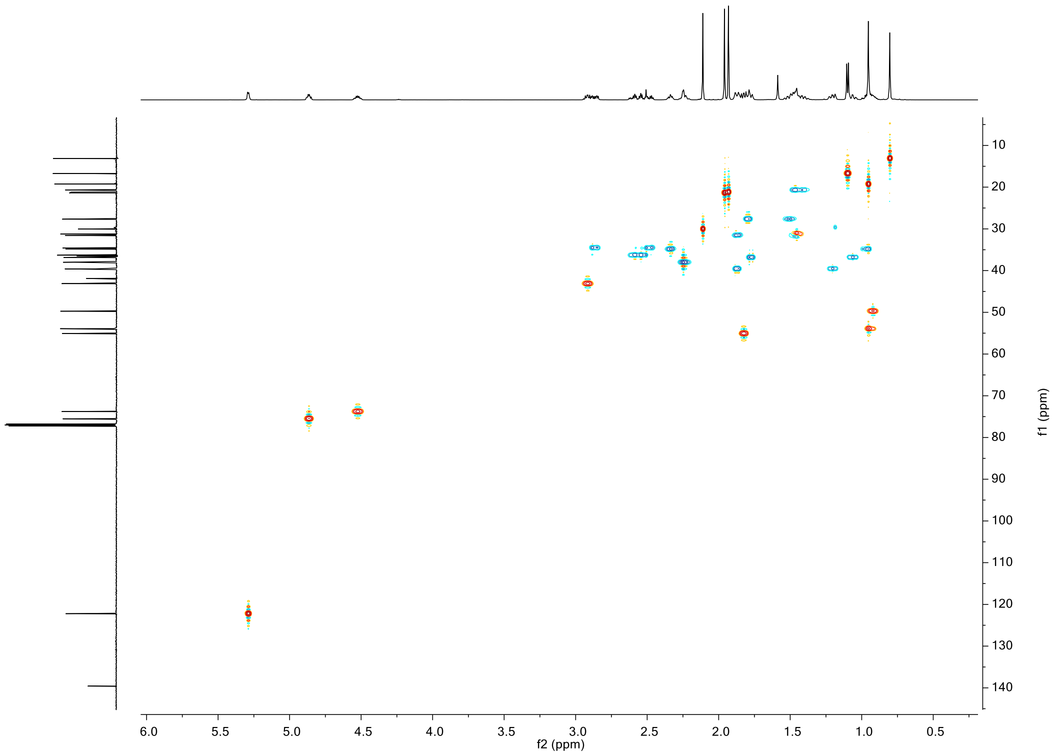


**Figure S19**. HSQC experiment compound **7**.

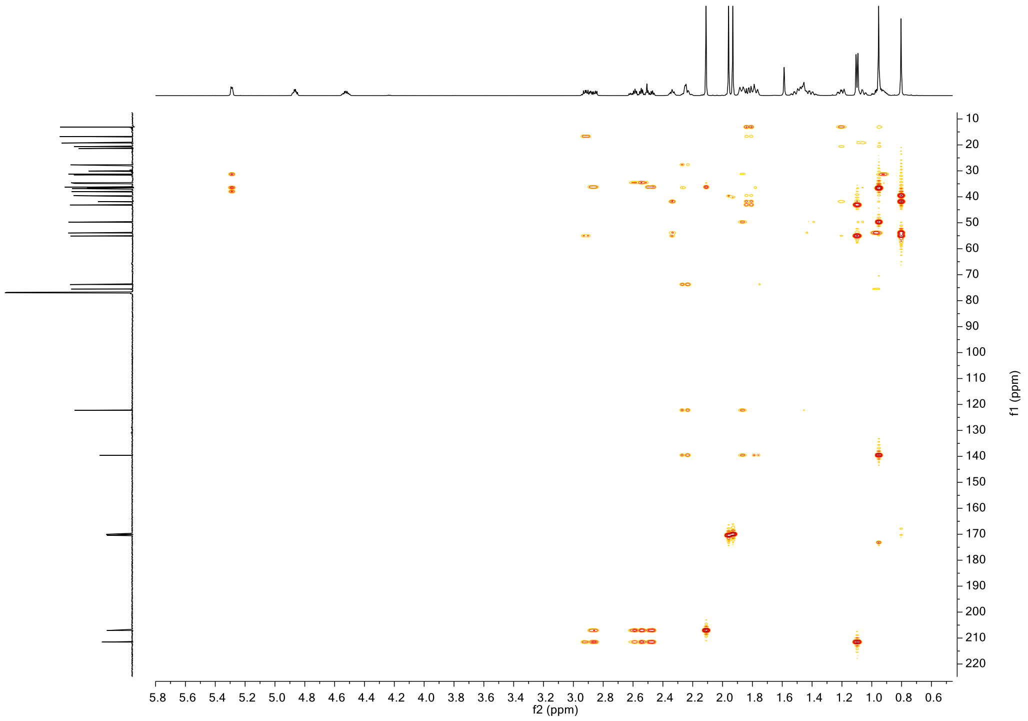


**Figure S20**. HMBC experiment compound **7**.

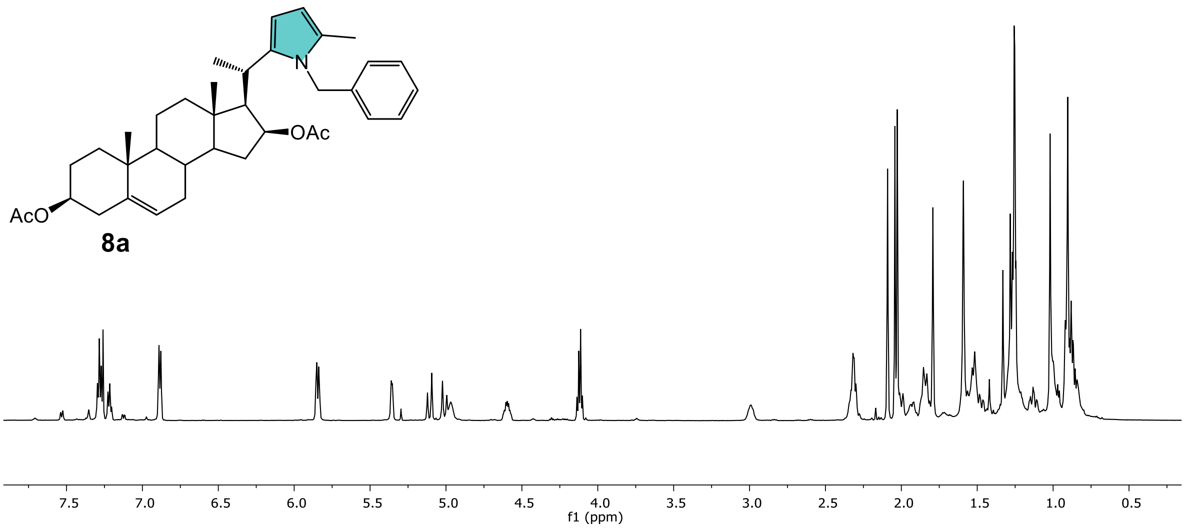


**Figure S21**. $^{1}$H NMR $CDCl_3$, 600 MHz compound **8a**.

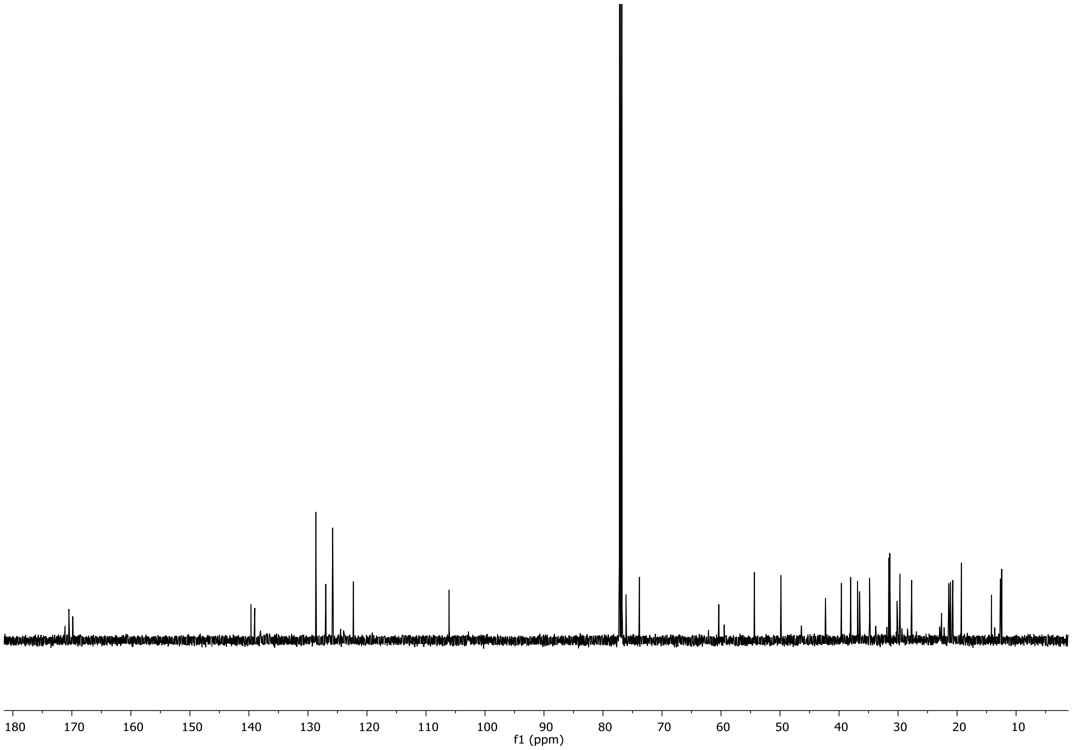


**Figure S22**. $^{13}$C NMR $CDCl_3$, 150 MHz compound **8a**.

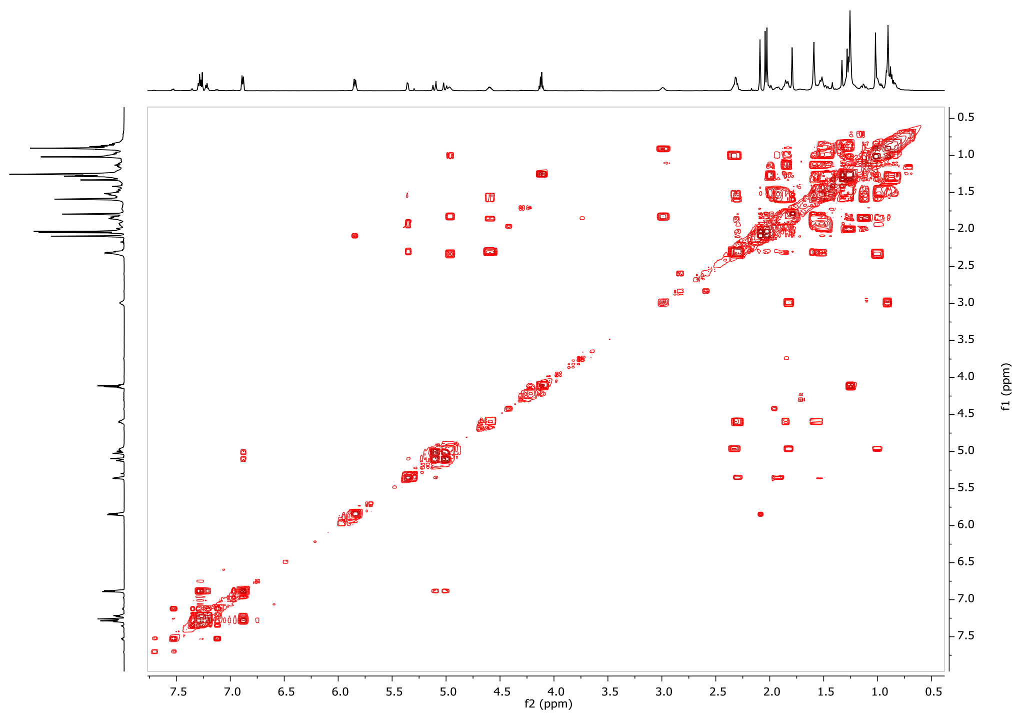


**Figure S23**. COSY experiment compound **8a**.

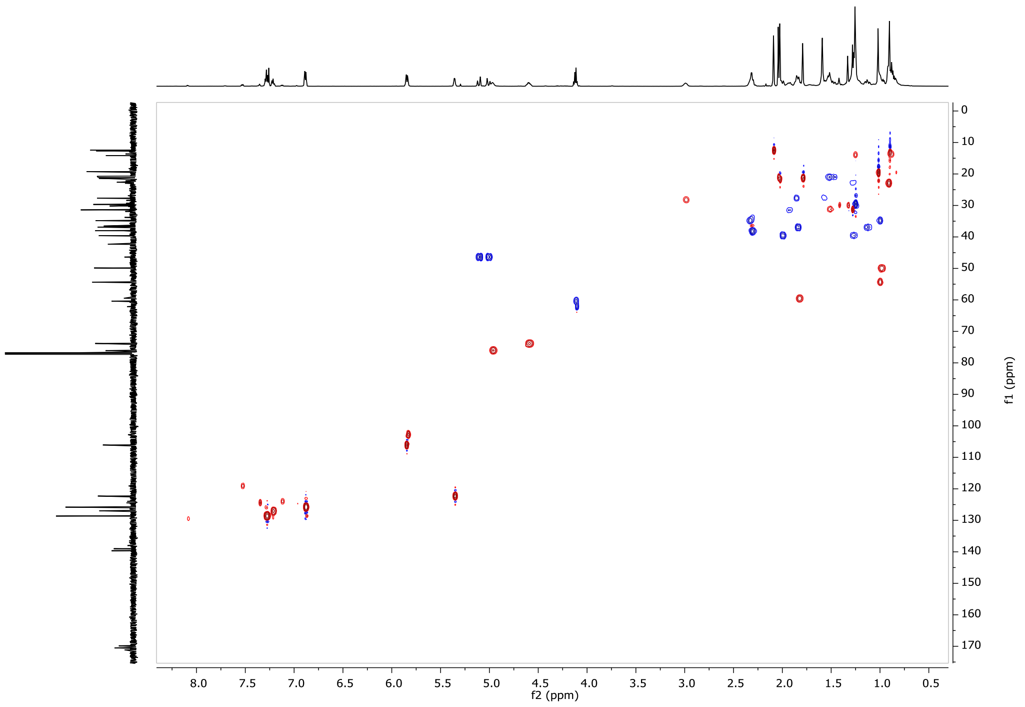


**Figure S24**. HSQC experiment compound **8a**.

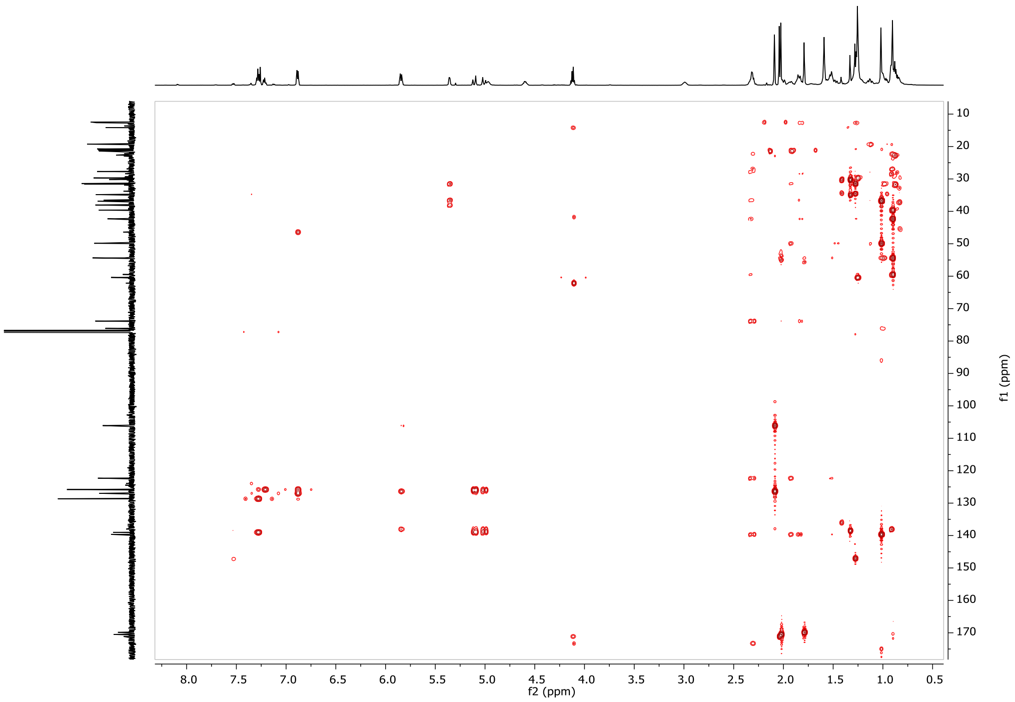


**Figure S25**. HMBC experiment compound **8a**.

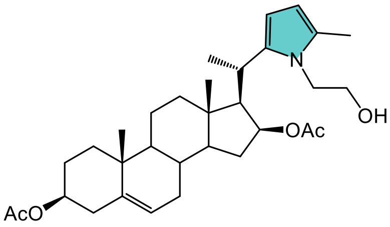


**8b**

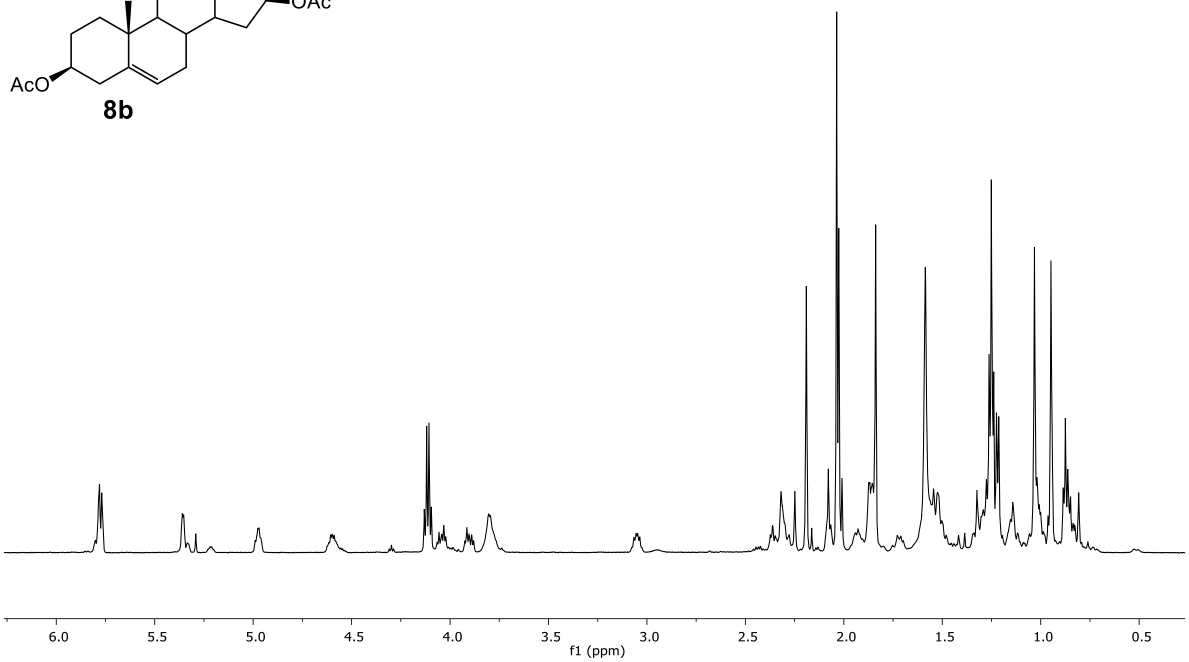


**Figure S26**. $^{1}$H NMR $CDCl_3$, 600 MHz compound **8b**.

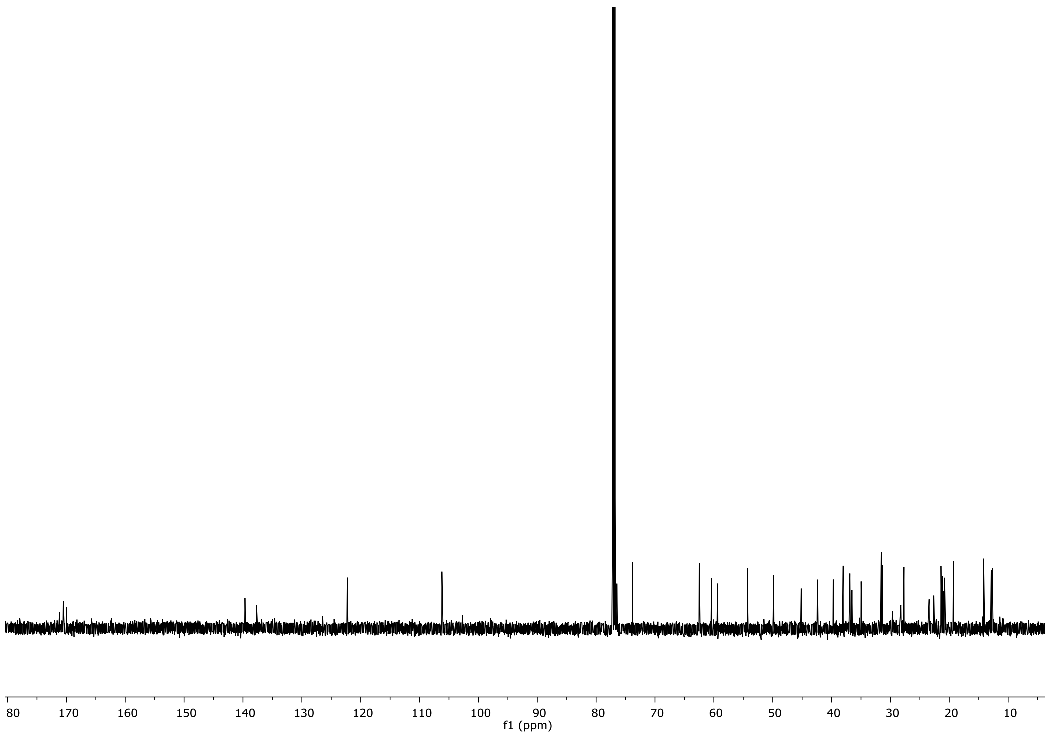


**Figure S27**. $^{13}C$ NMR $CDCl_3$, 150 MHz compound **8b**.

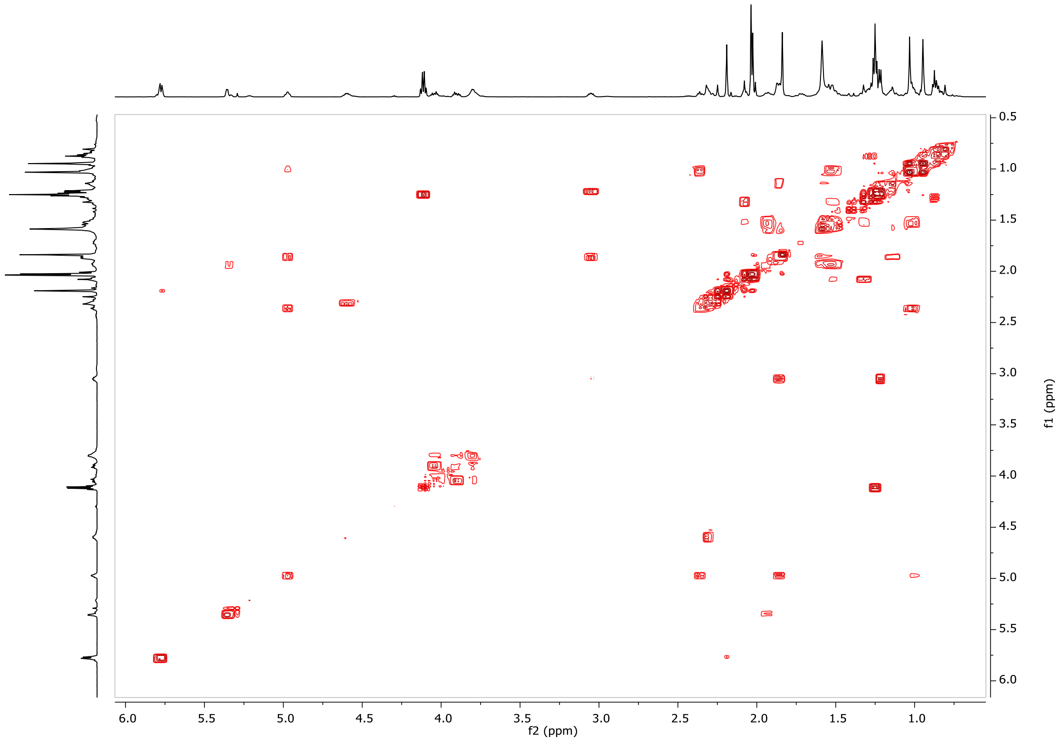


**Figure S28**. COSY experiment compound **8b**.

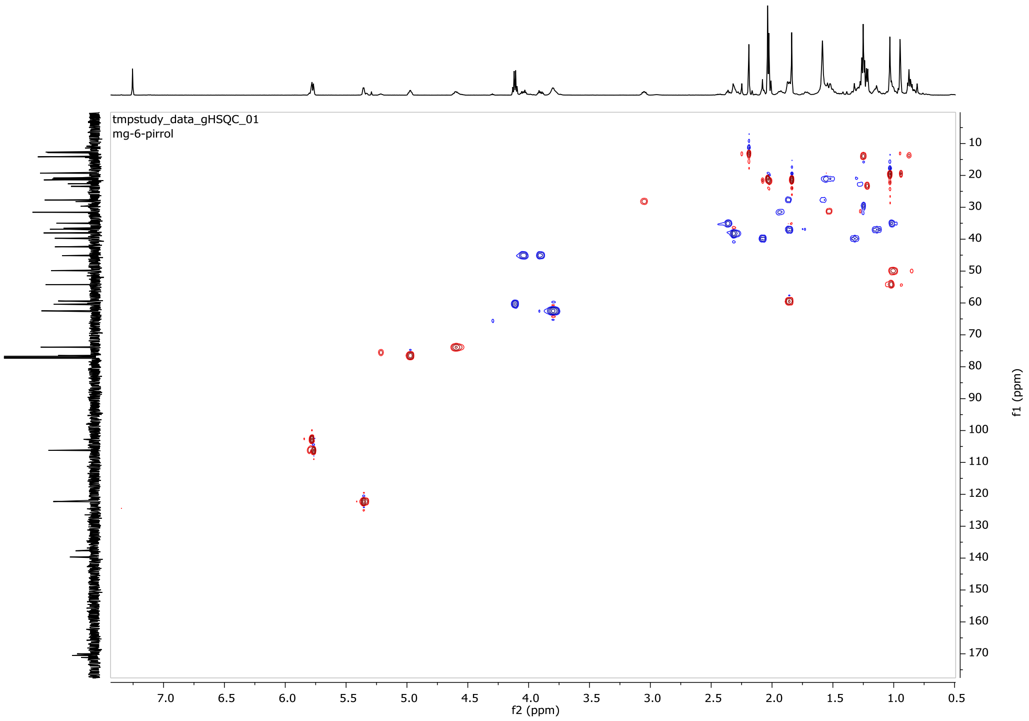


**Figure S29**. HSQC experiment compound **8b**.

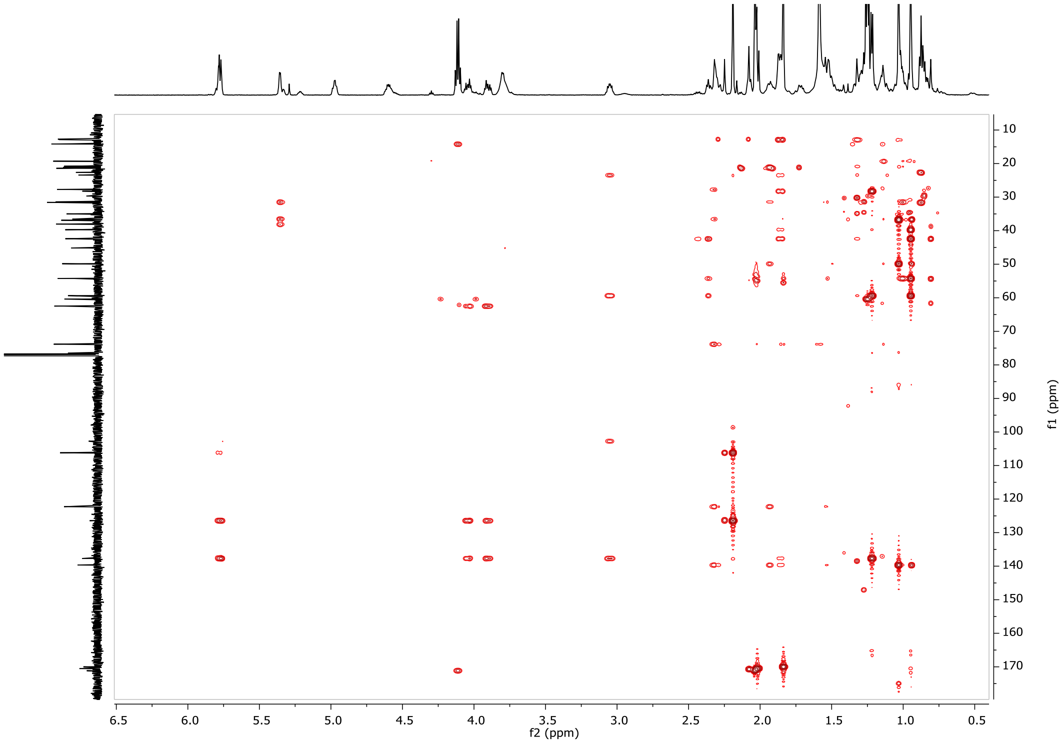


**Figure S30**. HMBC experiment compound **8b**.

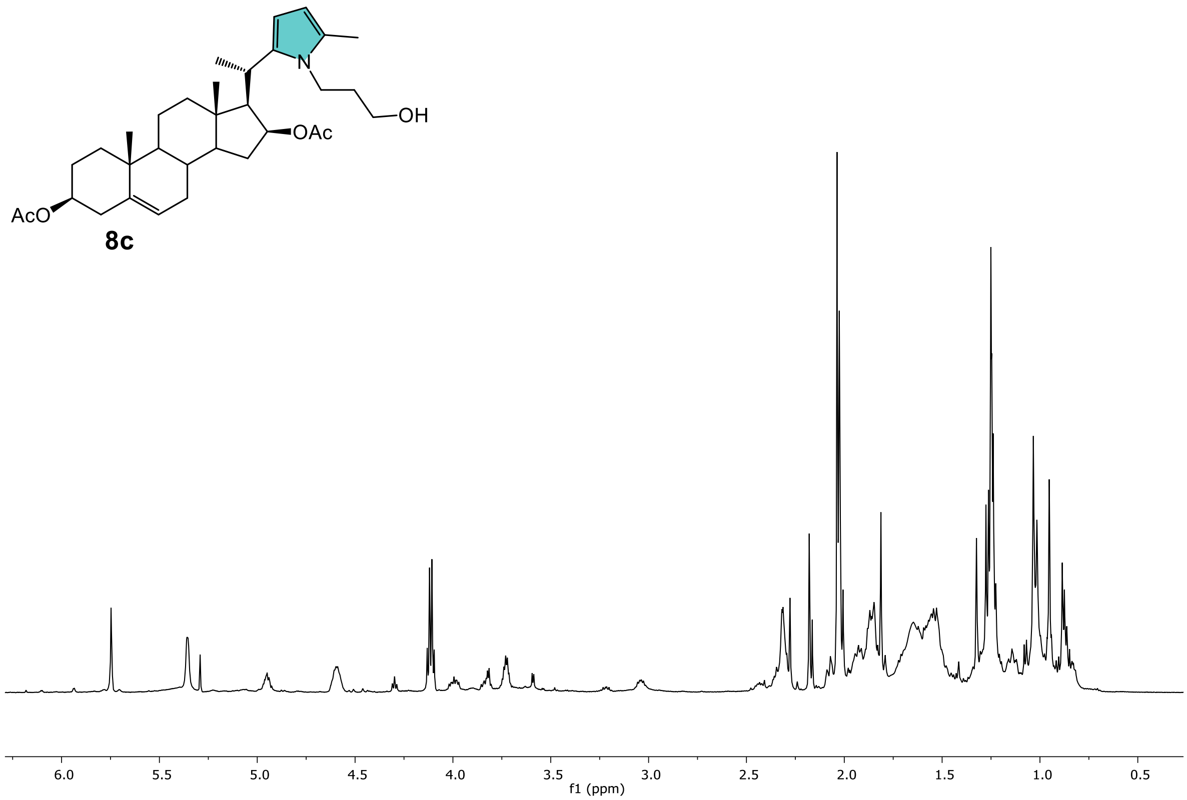


**Figure S31**. $^{1}H$ NMR $CDCl_3$, 600 MHz compound **8c**.

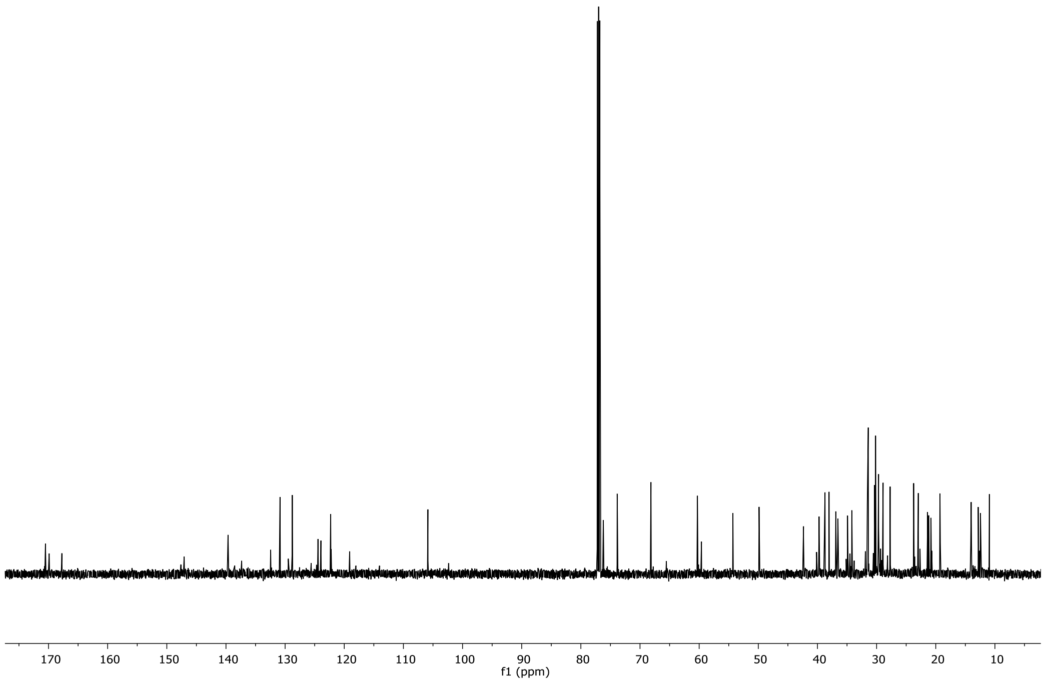


**Figure S32**. $^{13}C$ NMR $CDCl_3$, 150 MHz compound **8c**.

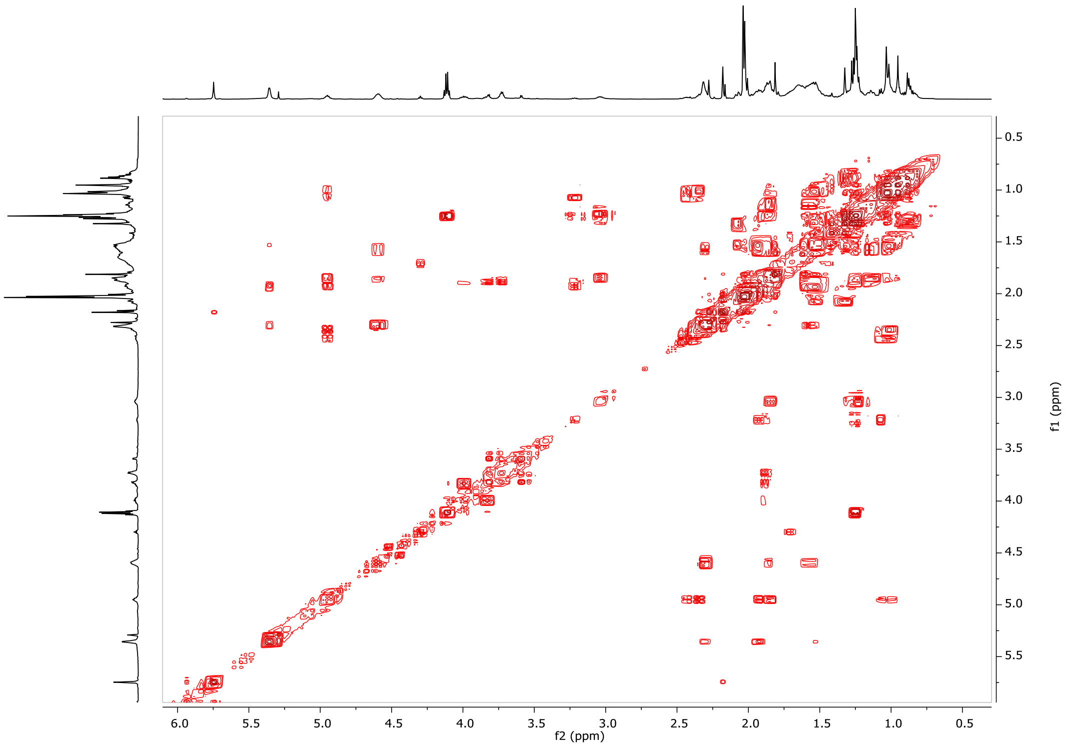


**Figure S33**. COSY experiment compound **8c**.

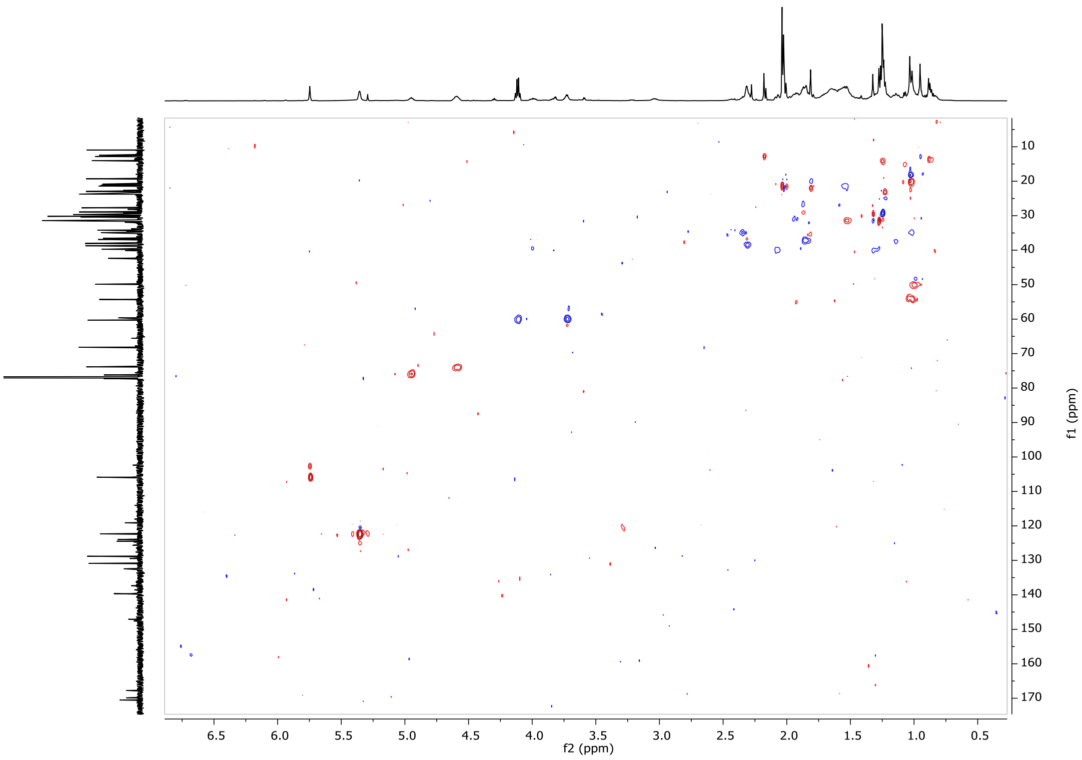


**Figure S34**. HSQC experiment compound **8c**.

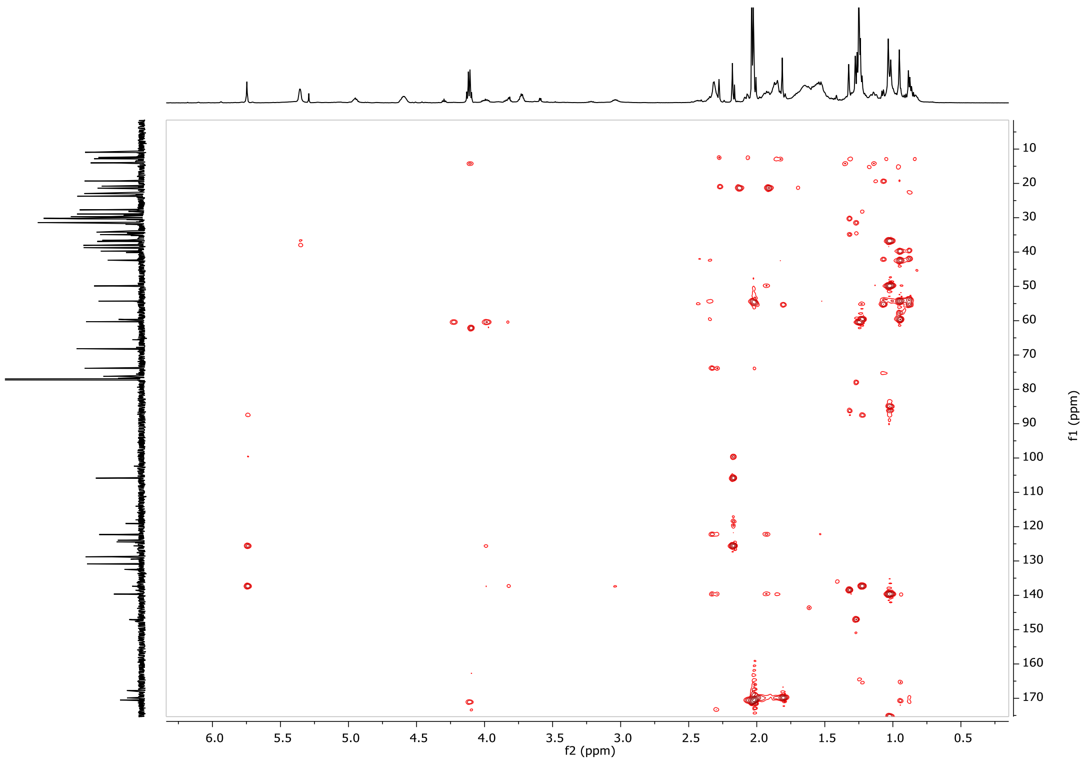


**Figure S35**. HMBC experiment compound **8c**.
